# Green quantification of amino(poly)phosphonates using ion chromatography coupled to integrated pulsed amperometric detection

**DOI:** 10.1007/s00216-025-05747-w

**Published:** 2025-01-28

**Authors:** Anna M. Röhnelt, Philipp R. Martin, Robert G. H. Marks, Daniel Buchner, Joachim Weiss, Torsten C. Schmidt, Stefan B. Haderlein

**Affiliations:** 1 Center for Applied Geoscience, Department of Geosciences, Eberhard Karls University Tübingen, Tübingen, Germany; 2 Instrumental Analytical Chemistry, University of Duisburg-Essen, Essen, Germany; 3 Institute of Analytical Chemistry and Radiochemistry, Leopold-Franzens University Innsbruck, Innsbruck, Austria; 4 Present Address: Division for Environmental Geosciences, Centre for Microbiology and Environmental Systems Science of Vienna, Vienna, Austria

**Keywords:** Aminopolyphosphonates (APPs), AMPA, Glyphosate, Integrated pulsed amperometric detection (IPAD), Green analytical chemistry (GAC), Transformation products

## Abstract

Aminopolyphosphonates (APPs) are widely used as chelating agents, and their increasing release into the environment has raised concerns due to their transformation into aminomethylphosphonic acid (AMPA) and glyphosate, compounds of controversial environmental impact. This transformation highlights the urgent need for detailed studies under controlled conditions. Despite the availability of various methods for quantifying individual aminopolyphosphonates and aminomonophosphonates, a green, low-cost approach for the simultaneous quantification of APPs and their transformation products in laboratory experiments has been lacking. In this study, we present a novel analytical method utilizing ion chromatography (IC) coupled to integrated pulsed amperometric detection (IPAD) to simultaneously quantify the six aminophosphonates: AMPA, glyphosate, iminodi(methylene phosphonate) (IDMP), aminotrismethylene(phosphonates) (ATMP), ethylenediamine tetra(methylene phosphonate) (EDTMP), and diethylenetriamine penta(methylene phosphonate) (DTPMP). This method achieves separation within a 35-min run time and method detection limits (MDLs) ranging from 0.014 μM for AMPA to 0.14 μM for DTPMP. The method’s applicability was successfully shown by monitoring DTPMP, IDMP, and AMPA during DTPMP transformation on manganese dioxide. A key advantage of this method is its environmental friendliness compared to existing aminophosphonate quantification techniques. Next to the simultaneous analysis, it avoids the use of derivatization agents and organic solvents and employs an energy-efficient detector. While the method’s limitations lie in the detector’s inherent non-specific nature, it offers a low-cost and sustainable alternative to existing methods.

**Graphical Abstract:**

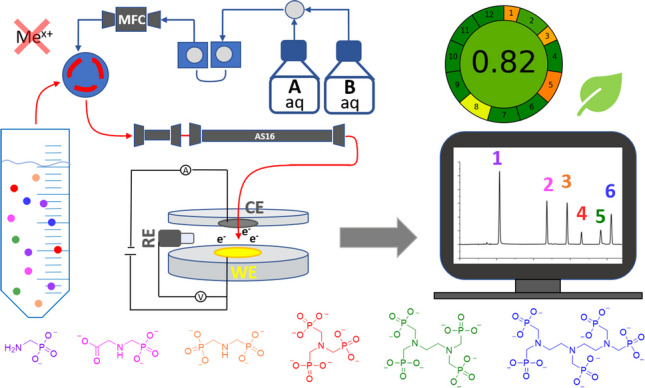

**Supplementary Information:**

The online version contains supplementary material available at 10.1007/s00216-025-05747-w.

## Introduction

### Aminopolyphosphonates

Aminopolyphosphonates (APPs) are strong chelating agents for di- and multivalent cations that are used in many household and industrial applications. The most important APPs by quantity are ethylenediamine tetra(methylene phosphonate) (EDTMP) and diethylenetriamine penta(methylene phosphonate) (DTPMP) [1]. APPs are constituents of, e.g., cleaning and bleaching agents, and are used as scale inhibitors in water treatment [1, 2]. Global phosphonate consumption was at 94,000 t in 2012—with 49,000 t thereof in Europe as reported by the European phosphonate association [1, 3]. The German “Industrial Association for Personal Care and Detergents” (IKW) [4] stated the total German phosphonate use in washing, care, and cleaning products with 7613 t/a in 2019.

**Fig. 1:**
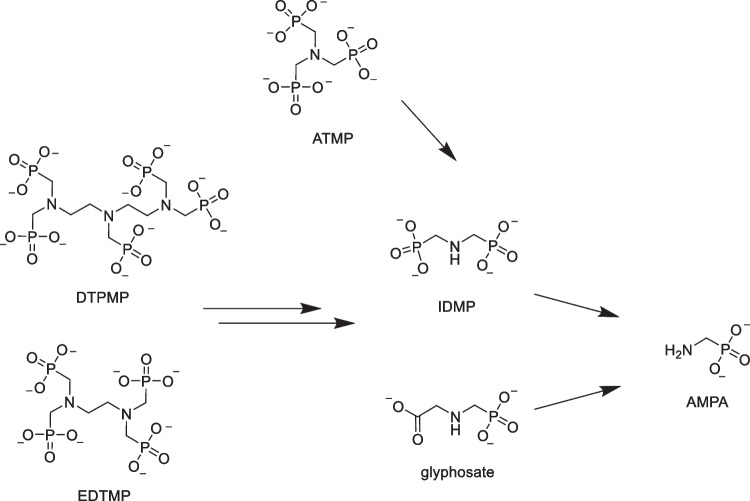
Fully deprotonated structures of DTPMP, EDTMP, and ATMP and their transformation products IDMP, AMPA and—in case of EDTMP and DTPMP—glyphosate

While phosphonates (including aminophosphonates (APs)) are generally assumed to be mainly removed from wastewater by adsorption onto sewage sludge [1, 5, 6], transformation of APPs under conditions relevant for environmental and technical systems is well described in literature, too. Studied reactions include (i) oxidation of ATMP in the presence of Mn^II^ and oxygen [7], (ii) oxidation of ATMP at MnOOH [8, 9], (iii) ozonation of EDTMP [10], and (iv) UV photolysis of free and complexed APPs [11–15]. The transformation products primarily include orthophosphate, aminomethylphosphonic acid (AMPA), and iminodi(methylene phosphonate) (IDMP). Furthermore, selected studies reported the minor formation of the controversially discussed herbicide glyphosate from EDTMP or DTPMP (see Fig. 1) [10, 16].

This suggests that household products may serve as a source of both glyphosate and AMPA, a conclusion that has been reinforced by recent studies which showed that WWTPs are sources of AMPA and glyphosate [17, 18]. Those observations emphasize the need to further investigate different APP transformation pathways. Such mechanistic studies call for well-designed laboratory batch experiments.

### Existing methods and greenness for polyphosphonate quantification

To investigate the transformation or sorption behavior of APPs in laboratory experiments, a green, easy, and low-cost quantification method is essential for the simultaneous quantification of APPs and their respective transformation products.

While green chemistry and atom economy are well-established tools in science and industry to sustainably design products and processes [19–21], the importance of green analytical chemistry has long been overlooked [22]. In the last years, green analytical chemistry (GAC) is increasingly gaining attention [23, 24]. Despite the efforts of several scholars to develop evaluation systems, the differences in purpose and the difference of the analytical methods itself for a long time made it quite difficult to derive exact “numbers of greenness” [20]. In the following, we refer to the 12 principles of GAC by Pena-Pereira et al*.* (2020) [24], who designed a general metric system and software to evaluate the greenness of analytical methods (AGREE).

Previous APP quantification methods generally do not perform well in AGREE, as they often require the use of a mass spectrometer (MS) [3, 25–28]. These MS-based methods are energy-intensive (and not cost-efficient), contravening principle 9 of GAC, “energy minimization.” Less expensive and less energy-intensive detectors can adequately monitor APP concentrations in, for instance, laboratory samples with a controlled matrix composition.

In addition, certain established methods require a pre-column derivatization with toxic compounds such as trimethylsilyldiazomethane or diazomethane [3, 27]. This process is time-consuming, introduces potential error sources, and generates toxic waste, violating principle 6 “Derivatization should be avoided,” principle 11 “Toxic reagents should be eliminated or replaced,” and principle 1 “Direct analytical techniques should be applied to avoid sample treatment.” As an example of nontoxic derivatization for non-MS methods, Fe^III^ is used for UV/Vis absorption detection, applied either post-column [29] or pre-column [30].

In general, APPs present significant analytical challenges, given their multiple negative charges, complex formation with bi- and multivalent cations [31–33], and the lack of chromophores or reactive groups [28]. Thus, IC methods using alkaline eluents without pre-column derivatization often exhibit suboptimal peak shapes, sometimes attributed to impurities in the phosphonate chemicals [30, 34, 35].

A compilation of published and validated APP quantification methods and their key parameters can be found in the SI (Table S1).

### AMPA and glyphosate quantification methods

The individual quantification of glyphosate and AMPA can be carried out with a variety of methods. Broadly used examples are (i) the use of liquid chromatography (LC) coupled to MS after derivatization with fluorenylmethyloxycarbonyl (FMOC) chloride [36–39], fluorescence detection after derivatization and separation using FMOC [40], or fluorescence detection after IC separation with post-column derivatization using *o*-phthaldialdehyde and Thiofluor® [41]. Described quantification methods without the need for derivatization are, for example, IC coupled to amperometric detection [42], capillary electrophoresis coupled to MS (CE-MS) [43], or hydrophilic interaction chromatography (HILIC) coupled to MS [14, 15, 44].

None of the methods published so far described the simultaneous quantification of glyphosate, AMPA, and IDMP together with ATMP, EDTMP, and DTPMP, which contrasts principle 8 “Multianalyte or multiparameter methods are preferred versus methods using one analyte at a time” [24].

### Amperometric detection

Electrochemical/amperometric techniques are promising in regards of greenness and specifically for the minimization of hazardous chemical usage [19]. For APs, amperometric detection is favorable in terms of low energy consumption but high sensitivity and selectivity [45]. The essential requirement for substances to be detectable via amperometry is their electroactivity, either given by aromaticity or the presence of oxidizable or reducible functional groups. For APs, this criterion is met by the presence of oxidizable hydroxyl and amino groups [46]. This allows for selective monitoring of APs and their electroactive transformation products. Compounds without electroactive groups, e.g., phosphate or methylene phosphonate, show no response and therefore cannot interfere with APP quantification [47].

Amperometric detectors comprise a three-electrode arrangement with a working electrode (WE), a counter electrode (CE), and a reference electrode (RE). The electrochemical reaction takes place at the WE, which is usually made of platinum or gold. In DC amperometry, a constant working potential is applied, by which the analytes are oxidized/reduced, and the resulting current is measured. However, for analytes precipitating or strongly sorbing at the electrode, constant working potential amperometry is not applicable due to baseline drift, increased background noise, and a constantly changing electrode surface resulting in a changing response [48]. Developments regarding the applied potential sequence (waveform) led to “pulsed amperometric detection” (PAD), usually involving a gold WE [48]. In contrast to amperometry with a constant working potential, PAD involves the repetitive application of a short potential sequence, typically lasting < 1 s, including a high oxidation and a low reducing potential. This potential sequence impedes electrode fouling or in other words “facilitates electrochemical cleaning” of the electrode surface in preparation for the next measurement interval. For further details on PAD waveforms, see the Supplementary Information.

Especially, developments in the field of amino acid detection using integrated pulsed amperometric detection (IPAD) [45, 47] and an IC-IPAD application note for glyphosate and AMPA with detection limits (LODs) below 2 μg/L [42, 49] suggested the suitability of IPAD for the quantification of APPs in aqueous solutions.

### Integrated PAD (IPAD)

IPAD is a variant of PAD. The waveforms developed for IPAD allow the simultaneous oxidation of the electrode surface and the analyte, also known as “mode II detection” [45–47]. The different potentials applied in IPAD are explained in Table S2. IPAD is predominantly used for the detection of amino acids, amines, and organic sulfur compounds. Their oxidation on metal electrodes is catalyzed by metal oxide formation [46]. While in pulsed amperometry the Faraday current is measured at a fixed oxidation potential, the integration part of the potential waveform in IPAD can have the form of a triangle or trapeze (see Figure S1 **b**), i.e., it is generally alternated between a high and a low potential [48]. While the surface oxide formation is necessary to catalyze analyte oxidation, the oxide formation produces a current itself—leading to high background currents.

In addition, concentration gradients are also the origin for high background currents caused by changes in the background electrolyte concentration throughout the gradient run [45, 47].

When IPAD is employed, baseline disturbances caused by pH gradients, ionic strength variations, and metal oxide formation are minimized. Because the oxidation of the electrode surface is a reversible process, while the oxidation of analytes is not, the resulting signal is mainly characterized by the contribution of the analyte oxidation. When integrating the current yield during the cycle, the net signal for the respective analyte is obtained [45, 47, 48, 50].

The oxidative detection of amino acids using IPAD is reported to be most effective on gold electrodes at strong alkaline pH [45, 47]. This provided the rationale for coupling anion-exchange chromatography with amperometric detection using a gold WE, employing eluents in the pH range of 11–13. Despite the sensitivity of this method to trace amounts of metal cations in the system due to strong complex formation with higher APPs, the alkaline pH range was chosen to simultaneously analyze aminomono, -bi-, and -polyhosphonates.

### Aim of this work

In summary, existing methods for the quantification of APPs have significant limitations concerning their greenness and especially the number of analytes that can be analyzed in a single chromatographic run. To address these shortcomings, a green and low-cost method for the simultaneous quantification of six APPs was developed. We describe here the systematic development and evaluation of an IC-IPAD quantification method, offering a cost-efficient, green, and sensitive approach. The applicability of the method will be demonstrated by monitoring the above-mentioned analytes in a DTPMP transformation experiment.

## Experimental section

### Chemicals

DTPMP (a) and EDTMP (b) have been purchased as solid acids from Zschimmer and Schwarz (Lahnstein, Germany) under the names “Cublen D 900 GR” (a) and “Cublen ELC 950” (b) (CAS: 15827–60-8 (a) and 1429–50-1 (b)). To ascertain the purity of the purchased substances, ^31^P-{^1^H}-NMR measurements were conducted, showing a purity of > 98.6% for DTPMP and 96.6% for EDTMP (nuclear magnetic resonance spectroscopy (NMR) measurements and results are described in the Supplementary Information, Figures S2, S3, and S4). Glyphosate (≥ 98.0%, analytical standard), AMPA (99%, analytical standard), IDMP (≥ 97%) and ATMP (≥ 97.0%), 2-aminoethylphosphonic acid (2-AEP, 99%), methylphosphonic acid (MPA, 99%), editronic acid (HEDP, ≥ 95%), phosphonoacetic acid (PAA, 98%), and phenylphosphonic acid (PPA, 98%) were purchased as solids from Sigma-Aldrich (St. Louis, MO, USA).

Sodium hydroxide (NaOH) for eluent preparation and analyte desorption from the manganese dioxide was purchased as a 49–51% solution from Supelco (Merck, Darmstadt, Germany), while sodium acetate (NaOAc) was purchased from Chemsolute (Renningen, Germany). MES buffer (≥ 99%) and MnO_2_ (manganese^IV^oxide) for the DTPMP transformation experiments were purchased from Carl Roth (Karlsruhe, Germany).

The cation-exchange resin in proton form (Dowex™ 50W X8 200–400, ≥ 1.7 eq/L) used to treat the experimental samples was purchased from Roth (Karlsruhe, Germany).

The water used has been purified by an ultrapure water purification system (Barnstead, GenPure Pro, Thermo Fisher Scientific, Waltham (MA), USA) down to a conductivity below 0.06 μS/cm.

### Instrumentation

A 930 Compact IC Flex ion chromatograph (Metrohm, Herisau, Switzerland) was used, equipped with a high-capacity anion-exchange column (Dionex™ IonPac™ AS16, 2 × 250 mm), a suitable guard column (Dionex™ IonPac™ AS16, 2 × 50 mm) and a metal-free trap column (Dionex™ MFC 500, all from Thermo Fisher Scientific, Waltham, MA, USA). Some tests have also been carried out using the anion-exchange column Metrosep A Supp 18 (4 × 150 mm, Metrohm) with the respective guard column (4 × 5 mm). The column temperature was set to 30 °C. The MFC 500, if used, was inserted between the pulse damper and the six-port injection valve.

The amperometric detector cell with a Wall-Jet geometry was equipped with a gold working electrode, platinum counter electrode, and a palladium or Ag/AgCl reference electrode (all Metrohm, see Figure S5). All columns, the detector, and electrodes have been used in the commercial state without any modifications. The detector temperature was set to 35 °C. The dosing units for (i) sample uptake and (ii) concentration gradient were both an “800 Dosino” (Metrohm), with (i) 2 mL and (ii) 5 mL cylinder volume.

To prevent CO_2_ dissolution into the eluents, an overpressure of 0.4 bar N_2_ was applied to both eluent bottles (gas-tight plastic bottles, Metrohm). The concentration gradient was achieved by an HPLC pump and a dosing unit comparable to a syringe pump (dosino). The dosino doses a defined amount of eluent B into a mixing piece, where it is mixed with eluent A. The eluent mixture is then conveyed by the HPLC pump. In order to prevent CO_2_ to dissolve in eluent B while the liquid is trapped in the dosino, the dosino is emptied completely at the start of each run and then filled just with the amount needed for one run (3 mL).

In this work, all chromatograms are displayed in Ampere on the *y*-axis, although IPAD is often displayed in Coulomb. To convert from nA to nC, the value in nA can be multiplied by the integration time in seconds, which is 0.380 s in the optimized method presented in this work.

### Maintenance

Due to the highly concentrated eluents and resulting salt precipitation, the HPLC pump head (although a chemically inert PEEK pump head) was rinsed weekly with deionized water (flow rate of 2 mL/min for at least 15 min), disassembled, and manually cleaned every 2–3 months.

Bi- and multivalent cations need to be removed from the IC system and column. Therefore, the system including the analytical column was rinsed fortnightly using 0.05 M ethylenediaminetetra(acetic acid) (EDTA, ≥ 97%, p.a., Roth (Karlsruhe, Germany)) at pH 6.5 to chelate and remove Fe (flow rate 0.5 mL/min, 1.5 h). Additionally, great attention must be paid to the metal parts in the system which can corrode even if they are not in contact with the eluent because the smallest leaks (e.g., from scratches due to salt precipitation) allow Fe to enter the IC system and hamper the quantification of ATMP, EDTMP, and DTPMP. Therefore, the MFC 500 (metal-free trap column) is placed directly after the pulse damper, to trap any Fe that may leak from the pump head or the pulse damper. Fortnightly, the MFC 500 was regenerated with 20 mL 1.5 M HNO_3_ and then rinsed with eluent A for 30 min.

### Cyclovoltammograms

The cyclovoltammograms have been recorded with the amperometric detector (Wall-Jet Cell) of the IC as described above, equipped with a gold working electrode, platinum counter electrode, and palladium reference electrode. The detector temperature was set to 35 °C. The sweep was performed from a minimum of − 0.35 V to a maximum of + 0.75 V with a sweep rate of 0.1 V/s and a range of 20 mA (cycle length: 22 s). The respective phosphonate was present at a concentration of 0.5 mM in a background solution of 0.1 M NaOH. The average of three consecutive sweeps was taken.

### Method validation

To validate the analytical performance, calibration standards of all APs were prepared in ultrapure water. For EDTMP and DTPMP, the pH was adjusted to a value of ≥ 5 in order to dissolve them. Due to observed analyte transformation at room temperature and under light, the standards were stored at –18 °C in the dark until analysis. The calibration ranged from 0.05 to 20 μM for each compound. For two standards (1 μM and 10 μM), ten replicates were measured consecutively to show repeatability. In order to validate the repeatability of the calibration, four calibration measurements have been performed on different days.

The method detection limits (MDLs) for all compounds were determined using the MDL procedure described by the US environmental protection agency (US EPA, Revision 2, 2016) [51]. At an S/N ratio of ~ 5, the respective standards have been prepared in deionized water and measured eight times (*n* = 8) consecutively; hence, the MDL could be calculated using the singe-tailed Student’s *t*-value with a confidence level of 99% and the standard deviation of the eight replicates.

### Design of DTPMP transformation experiments

The experiments have been conducted in 50-mL centrifugation tubes (polypropylene, Fisher Scientific, Waltham, MA, USA) in an anaerobic glovebox (N_2_ atmosphere) from MBRAUN (Garching, Germany). First, DTPMP stock solution, MES buffer solution, and ultrapure water have been purged with N_2_ for 1 h and were then transferred to the glovebox together with the solid MnO_2_. Afterwards, DTPMP, MES buffer, and water were mixed to yield concentrations of 1 mM DTPMP and 20 mM MES. After taking the timepoint zero aliquot, MnO_2_ (1 g/L) has been added to start the reaction. The sampling procedure described in Röhnelt et al. [16] was followed. In the end, there were two phases for each sampling point—the aqueous and the sorbed phase. The analytes were desorbed from the MnO_2_ residue, using 0.1 M NaOH and 0.1 M NaH_2_PO_4_ in the ultrasonic bath for 30 min. After the desorption step, the desorbed analytes were in an aqueous phase again and are thus given in the unit μM, too. Samples were stored in the dark at − 20 °C until analysis. Prior to analysis, samples were defrosted, diluted 1:50, and treated with cation-exchange resin.

The aqueous and sorbed phases were quantified separately and the analyte concentrations summed after analysis (*c*_tot_ = *c*_aq_ + *c*_sorb_).

## Results and discussion

### Chromatographic separation

The separation of all six analytes (AMPA, Glyphosate, IDMP, ATMP, EDTMP, and DTPMP) on the Dionex™ IonPac™ AS16 column requires a concentration gradient, as already shown for some polyphosphonates with NaOH gradients [28]. This column is hydroxide-selective, i.e., it has been designed to be used with hydroxide mobile phases; thus, previous methods describe the use of pure hydroxide eluents for the separation of, e.g., perchlorate [52] and polyphosphates [53]. Yet, with the analytical setup used in this study, no satisfactory separation could be achieved with eluents consisting purely of NaOH. In addition, pure NaOH eluents up to a concentration of 120 mM result in a high background signal and comparably low sensitivity for IDMP, EDTMP, and DTPMP (see Figure S6). Therefore, sodium acetate (NaOAc) was tested as an alternative, as it has proven to be particularly suitable for eluting the more strongly retained analytes in ion chromatography of amino acids [54]. Furthermore, NaOAc is not electroactive and thus compatible with integrated pulsed amperometric detection.

Hence, eluent B was amended with NaOAc as the main eluting agent. Initial experiments have been carried out with a combined NaOH and NaOAc concentration gradient. However, increasing the NaOH concentration from 15 to 50 mM during the chromatographic run, a significant baseline shift due to the change in pH was observed. By applying a NaOAc concentration gradient while keeping the NaOH concentration constant at 15 mM, the baseline shift could be eliminated. Additionally, a significant increase in the analyte response of ATMP, EDTMP and DTPMP could be achieved. Figure S7 shows a comparison of the analyte response using a pure NaOAc gradient (0–400 mM) with constant NaOH concentration (15 mM) (**B**) and a combined NaOH (15–50 mM) and NaOAc (0–400 mM) gradient (**A**).

**Fig. 2:**
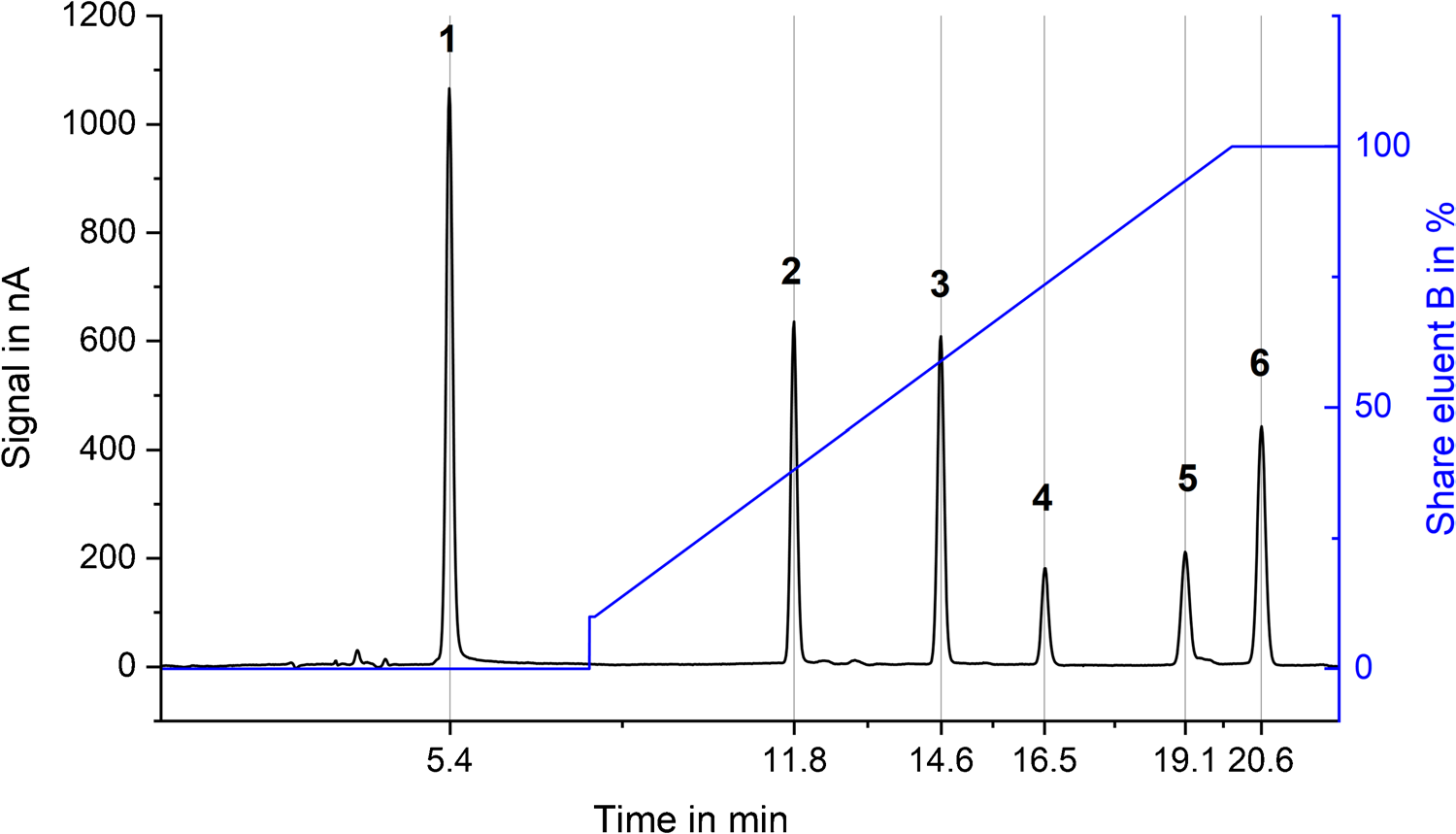
Optimized separation of a 10-μM multi-phosphonate standard using optimized chromatographic and amperometric parameters, shown after blank subtraction. Column: Thermo Scientific Dionex AS16 (2 × 5 + 2 × 250 mm) at 30 °C; MFC 500 inserted between pulse damper and six-port injection valve; eluents **A** 15 mM NaOH, **B** 15 mM NaOH + 400 mM NaOAc; flow rate: 0.3 mL/min; gradient profile: 0–6 min 0% B, 6–18 min 10–100% B, 18–21 min 100% B, 21.1–22 min 0% B, post run: 9 min with 100% eluent A at 0.6 mL/min; detection: amperometric detector with gold WE, Pt CE and Ag/AgCl RE, 35 °C; waveform: see Fig. 5 (**3**); injection volume: 50 μL; 10 μM of (1) AMPA, (2) glyphosate, (3) IDMP, (4) ATMP, (5) EDTMP, and (6) DTPMP

The optimized chromatogram was achieved by 15 mM NaOH (eluent A) and 15 mM NaOH plus 400 mM NaOAc (eluent B) and the gradient profile shown in Fig. 2. This chromatographic setup led to the elution order AMPA < glyphosate < IDMP < ATMP < EDTMP < DTPMP.

An inherent problem of NaOH eluents—manually prepared from NaOH concentrates—are carbonate impurities [55]. At low hydroxide concentrations, divalent carbonate ions accumulate at the stationary phase of an anion exchanger, thus reducing the anion-exchange capacity of the column over time, which results in a general RT decrease and a change in the elution order between IDMP and ATMP (Figure S8). Since analytes are identified via RT assignment, it is crucial to avoid carbon dioxide contamination in the eluent as much as possible to produce stable retention times. Therefore, several measures were taken to prevent CO_2_ contamination, such as N_2_ overpressure in the eluent bottles (see the “Experimental section”). With those measures in place reproducible RTs were obtained, with standard deviations ≤ 0.16 min (for DTPMP) and a maximum relative standard deviation of 1.3% for AMPA within 38 h (see Figure S9). However, over a longer period of time, a trend toward shorter RTs for all analytes was observed, which was compensated by the continuous measurement of external standards after six to ten sample runs (check standards). When strong retention time shifts were observed and two compounds were not separated to baseline anymore, the column was regenerated with freshly prepared NaOH (*c* = 300 mM) at a flow rate of 0.5 mL/min for 12 h.

**Fig. 3:**
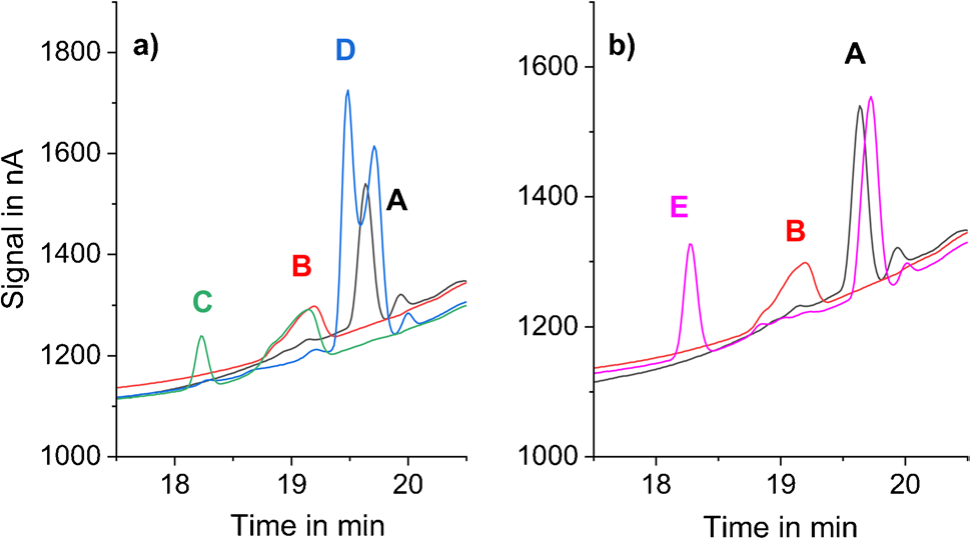
Chromatograms of EDTMP and DTPMP standards in a system with iron contamination. **a** (A) 10 μM DTPMP, (B) 10 μM EDTMP, (C) 20 μM EDTMP, (D) 20 μM, DTPMP. **b** (A) 10 μM DTPMP, (B) 10 μM EDTMP, (E) 10 μM EDTMP + 10 μM DTPMP. Chromatographic conditions: column: Thermo Scientific Dionex AS16 (2 × 5 + 2 × 250 mm) at 30 °C; eluents **A**: 15 mM NaOH, **B**: 50 mM NaOH + 400 mM NaOAc; flow rate: 0.3 mL/min; gradient profile: 0–6 min 0% B, 6–14 min 10–30% B, 14–18 min 30–100% B, 18–19 min 100% B, 20.1–22 min 0% B, post run: 8 min with 100% eluent A at 0.6 mL/min; detection: amperometric detector with gold WE, Pt CE, and Ag/AgCl RE, 35 °C; waveform: see Fig. 5 (**3**); injection volume: 50 μL

APPs form strong complexes with bi- and multivalent metal cations, such as copper and iron [32]. Therefore, bi- and multivalent cations need to be eliminated from the IC system and column. If metals are not eliminated, EDTMP and DTPMP show unreproducible chromatographic behavior, in the form of,e.g., two peaks or no peak in the chromatogram and/or distorted peak shapes, and therefore, their quantification is impaired. If Fe is present in the system, different Fe complexes can be formed depending on the analytes and analyte concentrations present in the standard/sample. Figure 3 depicts chromatograms of EDTMP and DTPMP influenced by Fe in the IC system. To achieve metal elimination, the system and column have been rinsed regularly with 0.05 M EDTA, and a metal-trap column has been inserted between pulse damper and six-port injection valve (see the “Experimental section” and “Maintenance” sections).

### Electrochemical detection

After the chromatographic separation, the detection parameters were optimized. A gold working electrode (WE) was chosen because of the strong adsorptive interaction between amines and the gold surface due to its unsaturated *d*-orbitals [47]. Platinum was chosen as counter electrode (CE, here: cathode) material due to its inert character. For the reference electrode (RE), palladium has been chosen as a starting point [42].

However, the majority of the aminophosphonate analytes under investigation are not readily oxidized between − 0.3 and + 0.75 V utilizing DC amperometry at the gold electrode (see cyclovoltammograms in Figure S10). Except for AMPA, the voltammograms of all other analytes (0.5 mM each) cannot be distinguished from the voltammogram of the background electrolyte (0.1 M NaOH). The peak in the positive scan in the cyclovoltammogram of 0.1 M NaOH corresponds to the formation of surface oxide at the gold electrode, while the peak in the negative scan corresponds to the reduction of surface oxide [46, 56].

Hence, APPs cannot be detected by applying the classical waveforms for pulsed amperometric detection (see Fig. 5 (**1** and **2**)) that was originally developed for the detection of carbohydrates [48]. On the other hand, the free electrons of the amine group in APPs can be oxidized utilizing IPAD waveforms on a gold working electrode.

#### Choice of the type of reference electrode

**Fig. 4:**
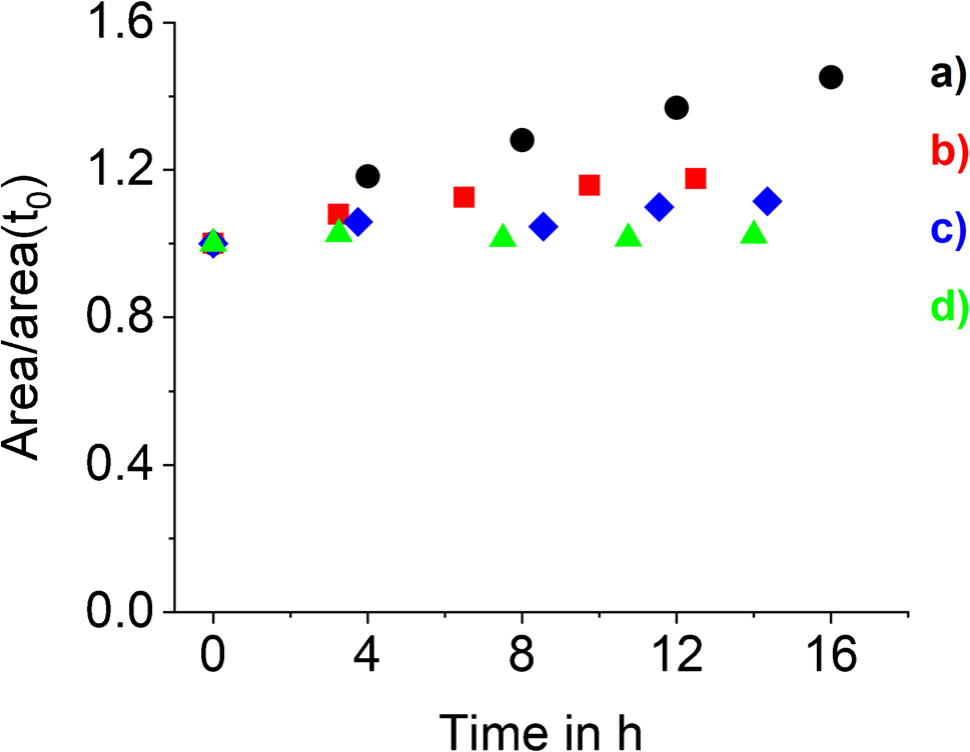
Normalized peak areas of glyphosate (10 µM) over time for different reference electrodes (RE) and potential waveforms. The used waveforms (**1**, **2**, and **3**) are depicted in Fig. 5. (a) Pd RE and waveform **1**, (b) Ag/AgCl RE and waveform **1**, (c) Ag/AgCl RE and waveform **2**, (d) Ag/AgCl RE and waveform **3**. Chromatographic parameters: column: Metrohm Metrosep A Supp18 (4 × 5 + 4 × 150 mm), 30 °C; eluents: A 20 mMNaOH, B 50 mM NaOH + 400 mM NaOAc; gradient profile: 0–3 min 5% B, 3–16 min 5–36% B, 16.1–18 min 50% B, 18.1–25 min 0% B. Detection: amperometric detector with gold WE and Pt CE; RE and waveform as denoted; detector temperature: 35 °C; injection volume: 50 µL

First, a Pd RE was utilized as it was already successfully used for IC-IPAD analysis of glyphosate and AMPA [49]. However, the electrode was not suitable for APP analysis as peak areas strongly increased for all six analytes (up to + 80% for EDTMP) within 32 h. As an example, Fig. 4a illustrates the peak area increase over time for 10 μM glyphosate. Insufficient cleaning of the working electrode was ruled out, as this would lead to a decrease in sensitivity. Therefore, it was hypothesized that the instable signal was caused by the sorption of APPs onto the reference electrode, altering the oxidation potential from the set value. To minimize the sorption of APPs an Ag/AgCl RE has been tested as described for EDTMP and DTPMP [34] and carbohydrates [55]. Using an Ag/AgCl instead of a Pd RE and applying the identical potential sequence, peak areas were significantly more stable (see Fig. 4b).

### Effect of IPAD wave forms

**Fig. 5:**
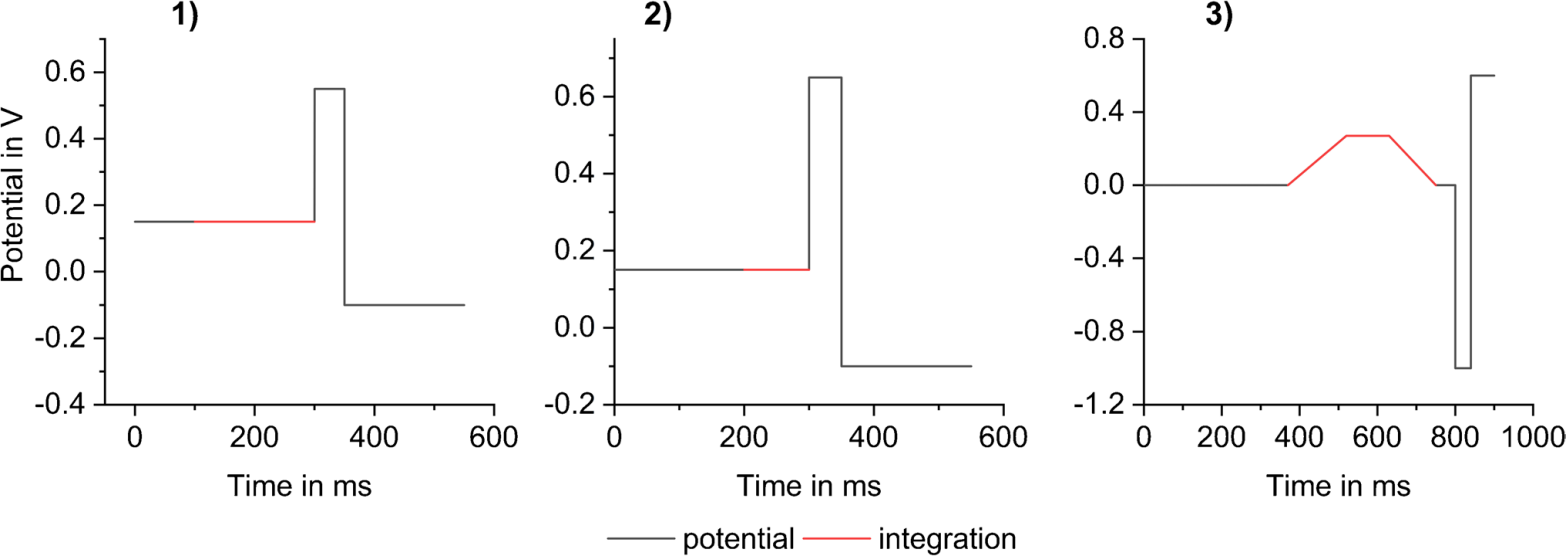
Waveforms used for the measurements presented in Fig. 4: (**1**) PAD with E_1_ = + 0.15 V, E_2_ = + 0.55 V, E_3_ = − 0.1 V and integration time of 200 ms, (**2**) PAD with E_1_ = + 0.15 V, E_2_ = + 0.65 V, E_3_ = − 0.1 V and integration time of 100 ms, (**3**) IPAD with E_1_ = 0.00 V, E_2_ = + 0.27 V, E_3_ = − 1.0 V, E_4_ = + 0.60 V and integration time of 380 ms; the exact potential sequence of (**3**) is presented in Table S4

IPAD is the method of choice for detecting amino compounds, including amino acids, due to the necessity of mode II detection [46]. Using IPAD together with the Ag/AgCl RE resulted in constant peak areas with marginal increases within 15 h (see Figs. 4d and 5 (3)). Additionally, the advantages of IPAD include lower background signal, a lower gradient-induced background increase when applying a concentration gradient, and increased analyte signals (peak areas) compared to tested PAD waveforms (see Fig. 5 (**1** and **2**)). Chromatograms recorded using PAD compared to IPAD are depicted in Figure S11.

To prepare the WE surface for the next oxidation reaction, a series of reduction and oxidation potentials are applied after every cycle like in standard PAD mode. The optimized IPAD waveform with a maximum detection potential (E_2_) of 0.27 V and an integration time of 750 ms is shown in Fig. 5 (**3**). This sequence resulted in low background signal, a high signal-to-noise ratio, symmetric peak shapes (see Fig. 2), and low method detection limits (MDLs) (see the “Method validation” section).

**Fig. 6:**
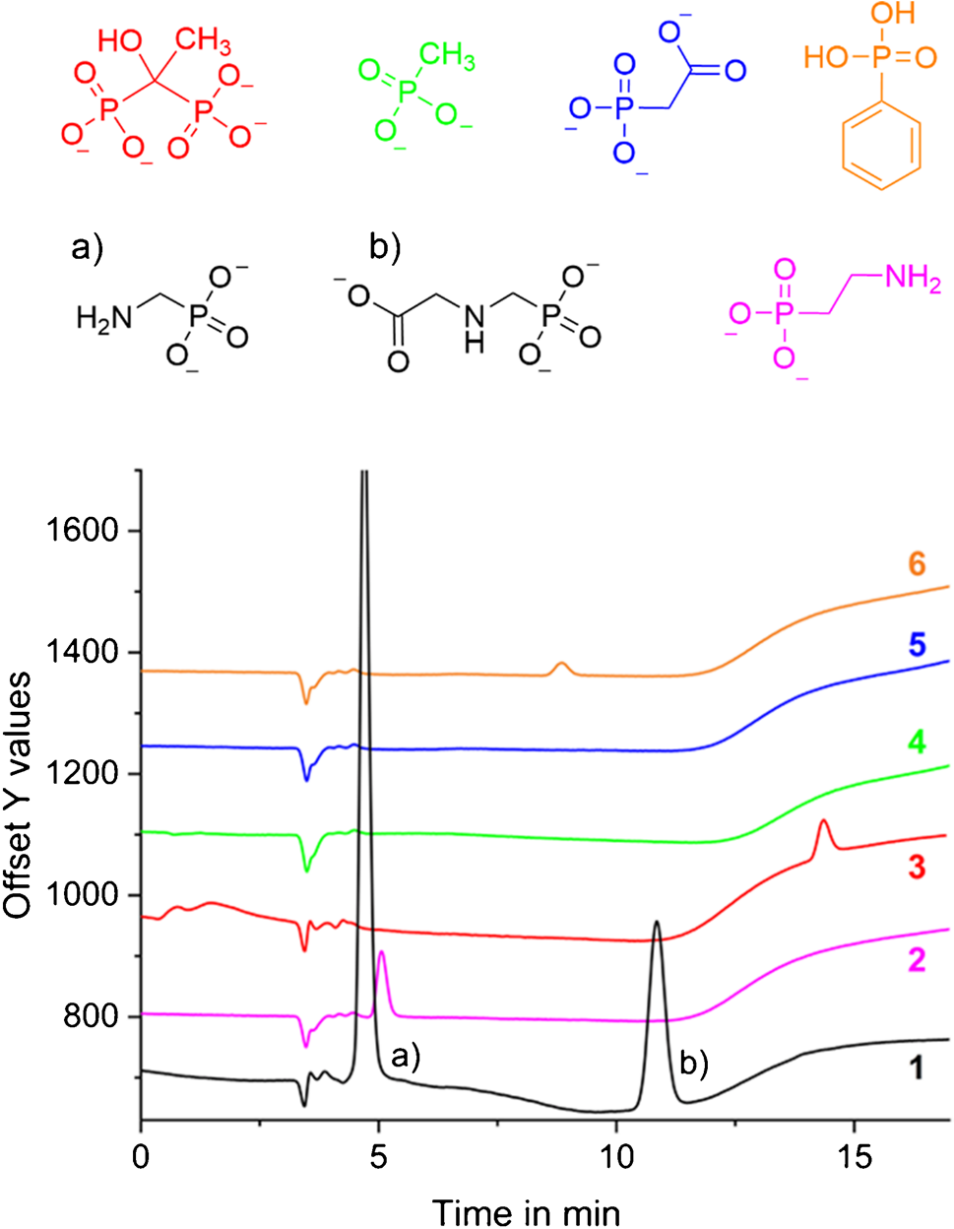
Chromatograms of seven different phosphonates. 20 μM of each compound: 1 = AMPA (a) and glyphosate (b), 2 = 2-aminoethylphosphonic acid (2-AEP), 3 = 1-hydroxyethylidene-1,1-diphosphonic acid (HEDP), 4 = methylphosphonic acid (MPA), 5 = phosphonoacetic, acid (PAA), 6 = phenylphosphonic acid (PPA). Chromatographic conditions: column: Thermo Scientific Dionex AS16 (2 × 5 + 2 × 250 mm) at 30 °C; eluents **A**: 15 mM NaOH, **B**: 50 mM NaOH + 400 mM NaOAc; flow rate: 0.3 mL/min; gradient profile: 0–5 min 10% B, 5–10 min 10–30% B, 10.1–15 min 0% B; detection: amperometric detector with gold WE, Pt CE and Ag/AgCl RE, 35 °C; waveform: see Fig. 5 (**3**) with E_2_ = 0.25 V

IPAD waveforms are described in literature to be very effective for amino compounds, but not showing a great response for alcohols or carbohydrates [46]. In order to assess which TPs can be detected using IC-IPAD, the phosphonate compounds 2-aminoethylphosphonic acid (2-AEP), etidronic acid (HEDP), methylphosphonic acid (MPA), phosphonoacetic acid (PAA), and phenylphosphonic acid (PPA) were tested. While two of them did not show any response at the before-mentioned detector (MPA, PAA), three of them showed a response (PPA, 2-AEP, HEDP), but one order of magnitude lower than the AMPA response: while 10 μM AMPA resulted in a peak area of approximately 260 nA*min, 20 μM HEDP (resp. 2-AEP, PPA) yield less than 10% of that (HEDP: 23 nA*min, 2-AEP: 27 nA*min, PPA: 7 nA* min, see Fig. 6).

Thus, the chosen detection method will primarily detect amino compounds with small signals for compounds bearing hydroxide groups or phenyl groups. The comparably low response of 2-AEP, which exhibits a primary amine function, needs further investigation.

### Method validation

All APs were calibrated in the range of 0.05–20 μM. Calibration curves were measured four times over 3 days to assess repeatability. Applying linear regression, the coefficient of determination (*r*^2^) showed values of ≥ 0.9960 for all six compounds. Over 3 days, the slopes of the linear calibration curves increased significantly, most pronounced for EDTMP and DTPMP, which is to be expected as the electrode surface is continuously altered throughout a measurement sequence. This underlines the need for continuous injection of check standards and/or new system calibration. However, the consistent high linearity of the single calibration curves proves the suitability of the method for external calibration.

To verify the repeatability of consecutively measured samples, the 1 μM and the 10 μM standard were measured eight times sequentially. The relative standard deviations (σ_r_) of the peak areas of those consecutive measurements were ≤ 5% (1 μM) and ≤ 3% (10 μM), respectively. The MDL was calculated according to the US EPA (Revision 2, 2016) using standards of the respective analyte with an S/N ratio of about 5.

Those analytical key figures are presented in Table 1. The peak width at half height was ≤ 0.13 min (glyphosate) for the 20 μM multi-standard. To assess the method’s analytical performance metrics, they will be compared with the figures of merit of other non-MS-based APP quantification techniques listed in Table S1.

**Table 1:** Analytical figures of merit of the optimized IC-IPAD method. σ_r_ denotes the relative standard deviation (n = 8) of the peak areas at the given concentration, *r*^2^ is the coefficient of determination for the linear regression of one standard curve in the given concentration range. MDL denotes the method detection limit

	AMPA	Glyph	IDMP	ATMP	EDTMP	DTPMP
*σ*_r_ (1 μM) in %	2.2	1.9	2.5	4.9	2.6	4.9
*σ*_r_ (10 μM) in %	1.0	0.8	1.0	1.7	0.3	2.3
*r*^2^ (0.05–20 μM)	0.9996	0.9991	0.9991	0.9997	0.9929	0.9987
MDL in μM	0.014	0.064	0.049	0.065	0.062	0.104

Nowack (1997) [30] reported detection limits (LODs) of 0.05 μM for ATMP and EDTMP, and 0.1 μM for DTPMP using pre-column Fe^III^ complexation and UV/vis detection. Weiss and Hägele (1987) [29] stated quantification limits (LOQs) in the lowest ppm range for ATMP, EDTMP, and DTPMP (single digit μM range). Tewari and van Stroe-Bieze (1997) [34], using amperometric detection, described their method as easily applicable to 25 mg/L (43.7 μM DTPMP), without stating explicit LODs.

The MDLs achieved in this study are comparable to those reported by Nowack (1997) [30], despite the use of a larger injection volume (200 μL) and broader DTPMP peaks (> 5 min width at 5 μM). Compared to the value provided by Tewari and van Stroe-Bieze (1997) [34], our method achieves MDLs approximately 100 times lower.

It is worth noting that these previous studies did not investigate method repeatability, which is crucial given the challenges associated with APP quantification [28, 57, 58]. Our detailed examination of repeatability and system maintenance provides valuable information for researchers in the APP field. The primary advantage of our method, however, lies in its green approach and simultaneous analysis of aminomono- and polyphosphonates, which will be discussed in subsequent sections.

### Greenness

To evaluate the greenness of the presented method in comparison to other published methods, the “Analytical GREEnness Metric Approach and Software” (AGREE) [24] has been used, which was recently employed (in an adapted version) in a number of studies [59–62]. This approach is based on the 12 principles of GAC and provides a score from zero (not green) to one (maximum green). Some aspects are briefly explained and conceptualized in the Supplementary Information to ensure transparency of the comparison.

For this comparison, the IC-ESI–MS quantification method published by Armbruster et al*.* in 2019 [28] and the LC-UV/vis method published by Nowack in 1997 [30] served as references. These methods were chosen, as both describe the analysis of the three APPs ATMP, EDTMP, and DTPMP. The IC-ESI–MS method represents a novel approach using mass spectrometry without derivatization. This allows for a comparison with a state-of-the-art technique. The LC-UV/vis method employs pre-column derivatization (complexation with Fe^III^), but no use of a mass spectrometer. By selecting these two methods, we can evaluate the greenness of our new IC-IPAD method against both a modern mass spectrometry-based technique and a simpler approach based on Fe^III^-APP complex formation.

**Fig. 7:**
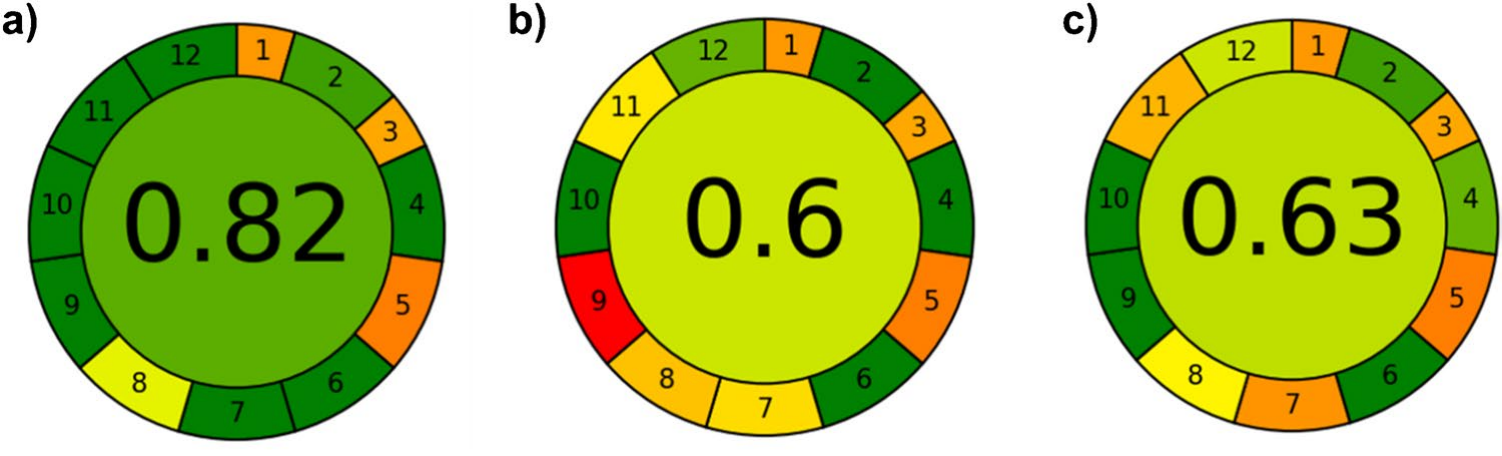
Greenness evaluation diagrams derived by the AGREE metric system and program from Pena-Pereira et al. (2020) [24] for **a** the IC-IPAD method presented in this work, **b** the IC-ESI–MS method published by Armbruster et al. (2019) [28], and **c** the LC-UV/vis method published by Nowack (1997) [30]

The IC-IPAD method (Fig. 7a) outperforms the other two methods (Fig. 7b and c) in several aspects. Regarding energy usage (principle 9), IC-IPAD consumes only about 0.035 kWh per sample, compared to over 1.5 kWh for IC-ESI–MS [24]. In terms of waste and toxic reagents (principles 7 and 11), the IC-IPAD method avoids the use of organic solvents like methanol or acetonitrile and the use of the ion-pair reagent tetrabutylammonium (TBA), which are used in the other methods [28, 30]. The sample preparation for the LC-UV/vis method requires a multi-step protocol, comprising cation exchange, Fe^III^-APP complexation, sequestration of excess Fe^III^ with NTA, and subsequent addition of a carbonate/TBA buffer. This elaborate procedure is time-intensive and contravenes the principles of integrating analytical processes (principle 4) and avoiding derivatization (principle 6). In contrast, the IC-IPAD method employs a less complex sample preparation protocol (solely cation exchange) and eschews the utilization of derivatization procedures.

Finally, the IC-IPAD method can simultaneously analyze six aminophosphonates, compared to three in the other methods (principle 8).

While the IC-ESI–MS method provides additional compound-specific information, this is not always necessary depending on the research requirements. The IC-IPAD method offers a good balance between analytical performance and greenness, making it particularly suitable for routine analysis of laboratory samples with known matrices.

It is important to note that the selection of an appropriate quantification method should be based on the specific research needs, and existing methods should be evaluated for potential improvements in greenness while considering these requirements.

### Application example: DTPMP oxidation by manganese dioxide

To demonstrate the suitability of the method described for laboratory APP transformation studies, DTPMP and its major electroactive TPs were quantified in the course of DTPMP oxidation by manganese dioxide. Measured concentrations over the course of the sequence were corrected using continuously measured check standards (1 μM, see Figure S12 and Table S5 for the correction function).

**Fig. 8:**
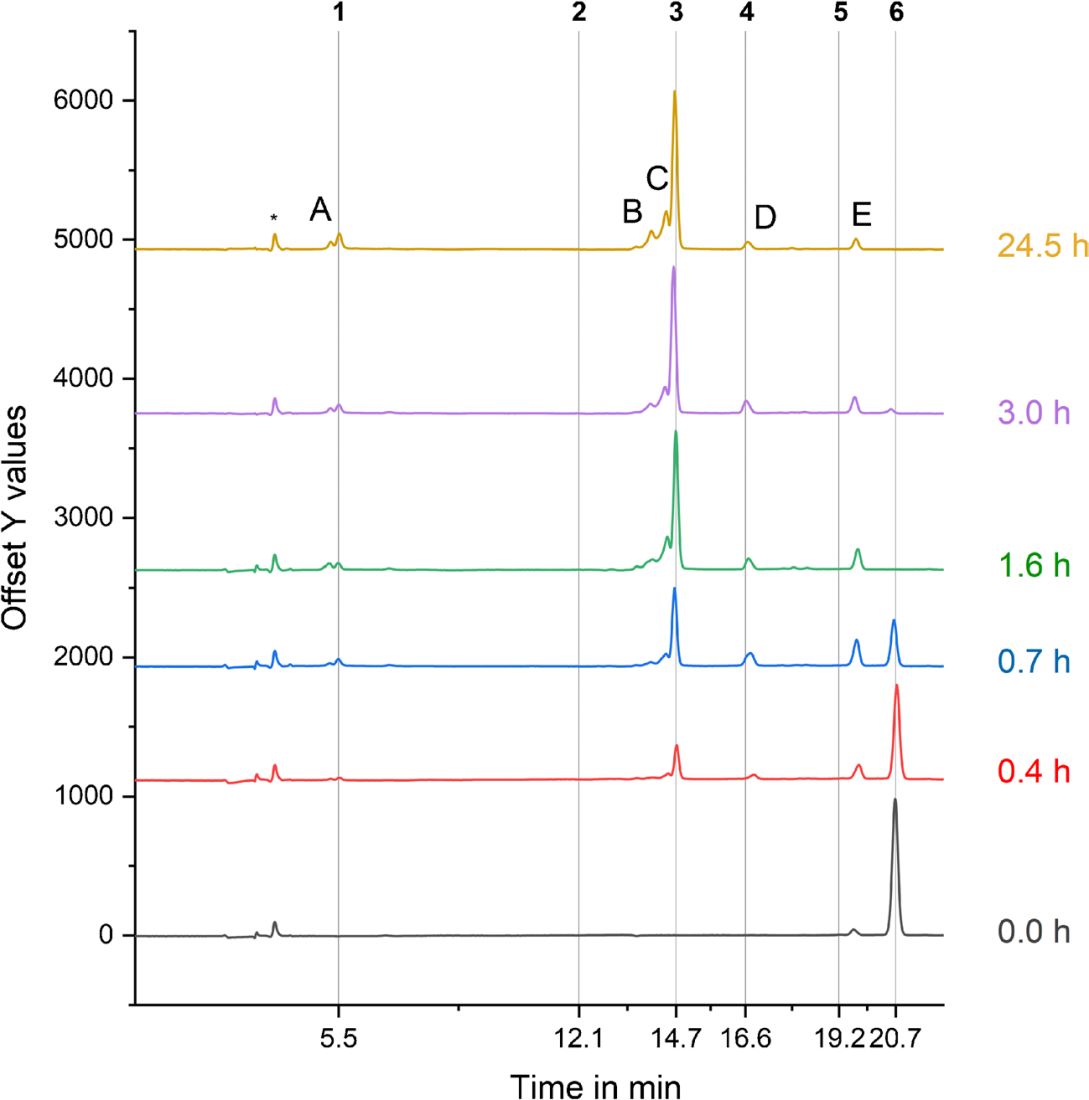
Stacked chromatograms of five different sampling points from the DTPMP transformation experiment (aqueous phase) with MnO_2_ shown after blank subtraction. The respective time in hours is denoted next to each chromatogram. The numbers denote the following compounds: (1) AMPA, (2) glyphosate, (3) IDMP, (4) ATMP, (5) EDTMP, (6) DTPMP. The asterisk denotes the injection peak. Unknown TPs formed are labeled with letters. Optimized chromatographic and amperometric parameters as described in the caption of Figs. 2 and 5 (**3**) were employed

Each sample was measured in triplicates. Figure 8 shows DTPMP transformation alongside the formation of TPs over time. While DTPMP concentrations decreased continuously, known (IDMP, AMPA) and unknown compounds appeared in the chromatogram; the latter were labeled with letters (see Fig. 8, 24.5 h). While some of the TPs (AMPA, IDMP) just increased, others decreased in concentration over the course of the experiment (D, E after 0.7 h) and, therefore, seem to represent intermediate products. For better depiction of the low concentrated analyte peaks, see Figure S13. DTPMP is almost completely transformed after 3 h. The similarity of the 3-h and 24.5-h samples demonstrates that the transformation products remain stable, with minimal changes occurring between these time points.

IDMP and AMPA were identified by RT assignment and standard addition (see Figures S14 and 15). However, the identification via standard addition is not a definite identification and thus needs to be verified by either plausibility or by identification methods such as IC-MS/MS. IDMP and AMPA are well-described as APP transformation products in literature [7, 12, 15] and are thus plausible and expected. In addition, AMPA was identified using LC–MS/MS [16]. Contrary, the positive result of the ATMP standard addition (Figure S15) illustrates the deficiency of this approach as the formation of ATMP (which would require a C-P bond formation) can be excluded in the conducted experiment. Thus, this peak represents a different compound coeluting with ATMP, probably also exhibiting 3–4 phosphonate groups.

**Fig. 9:**
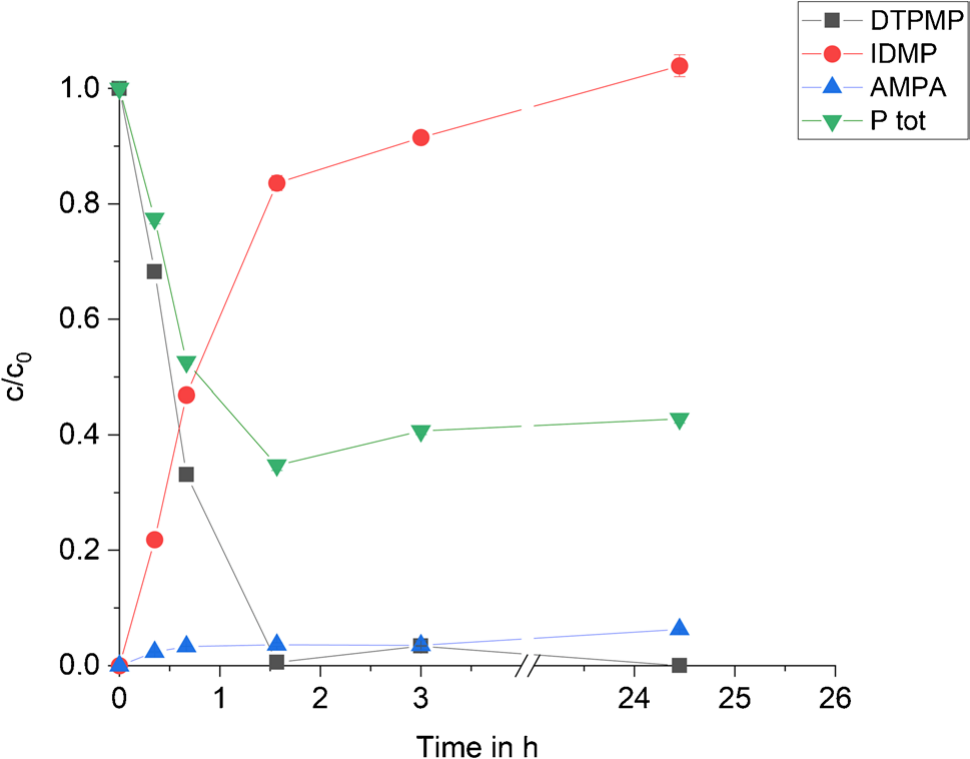
Total concentration profiles of DTPMP, IDMP, and AMPA normalized to the initial DTPMP concentration during DTPMP oxidation by MnO_2_ using 0.1 g/L MnO_2_ at pH 6 in an anoxic environment. “P tot” represents the phosphorus mass balance, which includes total P from all quantified compounds. Error bars represent standard deviations of triple measurements. Optimized chromatographic and amperometric parameters as described in the caption of Figs. 2 and 5 (**3**) were employed

Finally, the concentration profiles of the identified compounds DTPMP, IDMP, and AMPA versus time were plotted. Figure 9 shows the total concentrations (aqueous + sorbed) of the three compounds and the weighted phosphorus mass balance (*P*_tot_). The highest standard deviation was found for IDMP after 24.5 h (*σ* = 10.54 μM, *σ*_r_ = 1.1%). The weighted *P*_tot_ including DTPMP, IDMP, and AMPA amounts to 37.1 ± 0.5% at the end of the experiment.

Thus, the developed method (i) gives a good overview of the formed (electroactive) transformation products and (ii) allows the accurate quantification of TPs using external calibration with standards.

Other unknown transformation products showing a signal on the detector (A–E) could tentatively be identified by the respective standards and ultimately by high-resolution mass spectrometry (HRMS). For the determination of orthophosphate and other non-electroactive compounds, different analytical methods are required.

## Conclusion

The IC-IPAD method developed in this study represents a cost-effective and green method for the simultaneous quantification and monitoring of APPs and their transformation products in transformation studies. The method is suitable for the intended application of APP quantification in laboratory experiments and allows the monitoring of the environmentally relevant transformation products AMPA, glyphosate, and IDMP. This was demonstrated for a DTPMP transformation experiment with MnO_2_. MDLs between 0.014 μM (AMPA) and 0.104 μM (DTPMP) were achieved, while external calibration showed excellent linearity from 0.05 to 20 μM. This new method is superior in terms of environmental friendliness due to low energy consumption, elimination of any derivatization reactions and organic solvents in the mobile phase, and better simultaneous quantification of six analytes. However, unambiguous identification of unknown analytes and differentiation of coeluting compounds are not possible with this method due to the inherent limitations of the detection employed. If structural elucidation is required, the separation system could be hyphenated with tandem HRMS using a membrane suppressor system, while quantification could be performed using the method presented here. Future integration of green APP extraction methods could enhance the sensitivity of the method in complex environmental matrices, potentially extending its application range to natural samples.

## Supplementary Information

Below is the link to the electronic supplementary material.

Supplementary file1 The Supporting information contains further chromatograms, cyclovoltammograms, schemes, explanations, and data to underline the findings described in the main text. Further, it contains detailed information on the maintenance for the IC system and column. (PDF 1365 KB)
