# Supplementary material for: Green quantification of amino(poly)phosphonates using ion chromatography coupled to integrated pulsed amperometric detection
Source: Anal Bioanal Chem. 2025 Jan 28;417(8):1581–94. doi: 10.1007/s00216-025-05747-w (PMC11876216; doi:10.1007/s00216-025-05747-w)
Supplement: Supplementary file 1 — Supplementary file1 The Supporting information contains further chromatograms, cyclovoltammograms, schemes, explanations, and data to underline the findings described in the main text. Further, it contains detailed information on the maintenance for the IC system and column. (PDF 1365 KB) [file 216_2025_5747_MOESM1_ESM.pdf]

SUPPORTING INFORMATION

Green quantification of amino(poly)phosphonates using ion chromatography coupled to integrated pulsed amperometric detection

*Anna M. Röhnelt [1], Philipp R. Martin[1‡ *], Robert G. H. Marks[2], Daniel Buchner[1], Joachim Weiss[3], Torsten C. Schmidt[2] & Stefan B. Haderlein[1]**

Revised and resubmitted to: **Analytical & Bioanalytical Chemistry**

[1]Center for Applied Geoscience, Department of Geosciences, Eberhard Karls University Tübingen, 72076 Tübingen, Germany

[2]Instrumental Analytical Chemistry, University of Duisburg-Essen, Essen, Germany

[3]Institute of Analytical Chemistry and Radiochemistry, Leopold-Franzens University Innsbruck, Austria

[‡]Current address: Division for Environmental Geosciences, Centre for Microbiology and Environmental Systems Science of Vienna, Austria

Corresponding authors:

* stefan.haderlein@uni-tuebingen.de, philipp.martin@univie.ac.at

Table S 1: Compilation of published and validated APP quantification methods and their key parameters.

Reference	APPs	Column	Eluents	Derivatization	Detector
**Wang et al. 2019** (1)	DTPMP, EDTMP, ATMP, HDTMP (PBTC, HEDP)	LC (ZORBAX Eclipse Plus C18)	H_2O, ACN (0.1 % Formic acid)	Trimethylsilyl-diazomethane	HESI-MS/MS (QQQ)
**Fürhacker et al. 2005** (2)	DTPMP, EDTMP, ATMP	IC (Dionex AG7-AS7)	H_2O (1 % MeOH), 100 mM HNO3 (1 % MeOH)	-	ICP-MS (QQQ)
**Schmidt et al. 2014** (3)	DTPMP, EDTMP, ATMP, HDTMP (HEDP)	IC (Thermo IonPac AS16)	H_2O, NaOH, (addition of DTPA)	-	ICP-MS
**Armbruster et al. 2019** (4)	DTPMP, EDTMP, ATMP (PBTC, HEDP)	IC (Thermo IonPac AS16)	H_2O, NAOH, MeOH	-	ESI-MS/MS (QQQ)
**Klinger et al. 1997** (5)	DTPMP, EDTMP, ATMP, HDTMP (PBTC, HEDP)	LC (Merck LiChrospher 100 Diol)	Isopropyl alcohol, n-Hexane	Diazomethane	Particle beam (PB)-MS
**Tewari et al. 1997** (6)	DTPMP, EDTMP	IC (Thermo AS11)	0-200 mM NaOH	-	PAD (Au WE, Ag/AgCl RE)
**Wong et al. 1987** (7)	DTPMP, EDTMP, ATMP, HEDTMP (HEDP)	IC (Waters IC Pak A)	Nitric Acid (15 mM)	-	RID
**Weiss & Hägele 1987** (8)	DTPMP, EDTMP, ATMP (HEDP)	IC (HPIC AS7, Dionex)	30-70 mM HNO_3	Post-colµmn: Complex formation with Fe^{III}	UV/vis absorption
**Nowack 1997** (9)	DTPMP, EDTMP, ATMP (HEDP)	LC (ion-pair chromatography using RP colµmn: PLRP-S, polymer labs)	20 mM $NaHCO_3$ and 1 mM tetrabutylammonium bromide in H_2O	Pre-column: Complex formation with Fe^{III}	UV/vis absorption

**Vaeth, Sladek & Kenar 1987** (10)	ATMP, EDTMP, DTPMP	IC (HPIC AS7, Dionex)	3.2 mM EDTA + 0.17 mM KCl in H_2O, pH 5.1	Post-colμmn: oxidation to phosphate by persulfate, then derivatization using molybdenum blue	UV/vis absorption

Background on Pulsed Amperometric Detection (PAD)

In PAD, a short potential sequence including a high oxidation and a low reducing potential, typically lasting <1 second is repetitively applied. This is particularly advantageous when analytes and/or transformation products strongly adsorb onto the electrode surface and was primarily developed for the analysis of carbohydrates (11). After the oxidation step, a higher oxidation potential is applied to oxidatively remove residual analyte and/or transformation products from the WE. In the successive reduction step, a low reduction potential is applied to reduce the formed gold oxide back to gold. To prevent monitoring non-faradaic current, a delay time is required prior to the measurement interval, allowing charging currents to decay (11,12). A schematic depiction of a PAD waveform is provided in Figure S1 **a**. Developments regarding PAD and instrumentation allow this detector to be coupled to high-pressure liquid chromatography (HPLC) systems with purely aqueous eluents, despite the inherent sensitivity of pulsed amperometric detection toward pressure and pH.

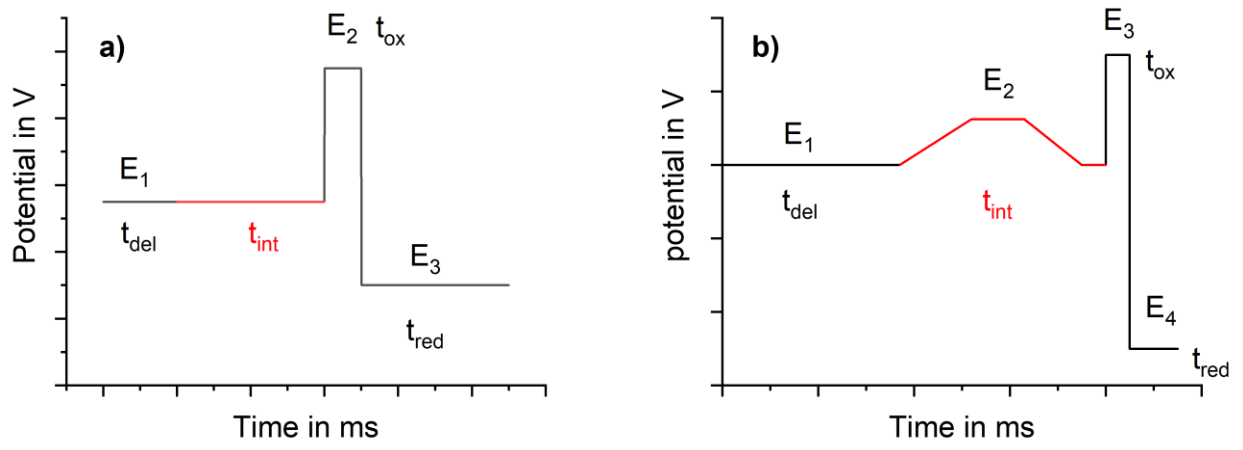

Figure S 1: Exemplary potential cycles of a) PAD (corresponding to mode I detection) and b) integrated PAD (corresponding to mode II detection).

Table S 2: Explanation of different potentials and times applied in the IPAD waveform (see Fig S1 ***b****) (11,13)*

**Potential/Time**	**Description**
E_1	Starting (prior to onset of oxide formation) and ending potential of the scan (more negative than oxide dissolution)
E_2	Maximum potential of the scan for optimal analyte oxidation
E_3	Oxidation potential to initiate formation of “cleaning oxide”
E_4	Reduction potential to initiate dissolution of inert oxide
t_{del}	Delay time: Allow the charging current to decay, before the faradaic current produced by redox reactions at the electrodes is recorded
t_{int}	Integration time: Measurement signal is recorded
t_{ox}	Time for application of the oxidation potential E_3
t_{red}	Time for application of the reduction potential E_4

Purity of DTPMP & EDTMP

<u>Nuclear magnetic resonance spectroscopy (NMR)</u>

To assure the purity of the acquired EDTMP, ^{1}H- and ^{31}P-{^{1}H}-NMR measurements were conducted (NMR department, Chemistry Department, University of Tübingen).

10 mg EDTMP (DTPMP) and 600 µL of deuterated water (D_2O) were mixed and vortexed for 5 s. Afterwards, 600 µL were transferred to an NMR glass tube. The measurement was performed on a Bruker AMX 600 MHz NMR spectrometer (Bruker, Billerica, MA, USA), operating at 600.13 MHz for hydrogen observation with a zg30 pulse program and at 242.94 MHz for phosphorous observation with a zgpg30 pulse program. The acquisition parameters used for this experiment with 1D sequence with power-gated decoupling and a 30 ° flip angle were as follows for ^{1}H (^{31}P): number of scans: 32 (64), spectral width: 12019.23 Hz (96153.84 Hz), offset (O1): 3705.80 Hz (-12146.85 Hz), acquisition time: 2.73 s (0.34 s), relaxation delay (d1): 1.00 s (2.00 s). The spectrum was quantitatively evaluated using the Bruker Top Spin 4.1.4 software.

Results

^{31}P-NMR-spectroscopy, due to its 100% natural abundance, wide range of chemical shift and high sensitivity(14), is a suitable analytical tool to characterize the purity of phosphonates.

EDTMP

In the ^{31}P-{^{1}H}-NMR-spectrum of EDTMP, shown in Figure S2, we can see one signal attributed to EDTMP representing all chemically equivalent phosphonate-groups (δ (ppm): 8.76) Impurities are marked with "*". The sum of all signal-integrals is normalized to 100. Impurities containing phosphorous of the analysed EDTMP amount to only 3.40 %. The used EDTMP in the experiments is therefore of high purity with 96.6 %. The ^{1}H-NMR-spectrum is shown in Figure 2.

Therein we can see the signals attributed to EDTMP in a ratio of 4:8. The singulet represents the protons of the ethylenediamine moiety in the middle (δ (ppm): 3.88) and the duplet represents the protons of the four phosphonate groups attached to the amine-moieties (δ (ppm): 3.52). Impurities are marked with "*". The sum of all signal-integrals is normalized to 100. Impurities of the analysed EDTMP amount to only 2.52 %. The used EDTMP in the experiments is therefore of high purity with 97.48 %.

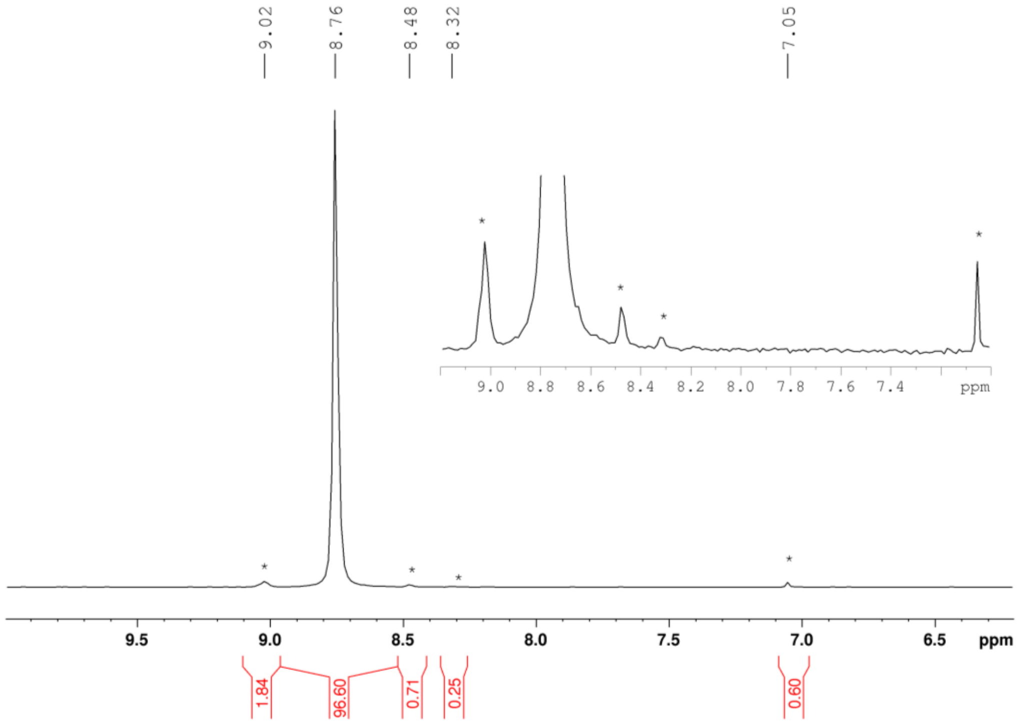

Figure S 2: ^{31}P-{^{1}H}-NMR-spectrum of EDTMP in D_2O. δ (ppm): 8.76. Impurities are marked with "". The sum of integrals is normalized to 100.*

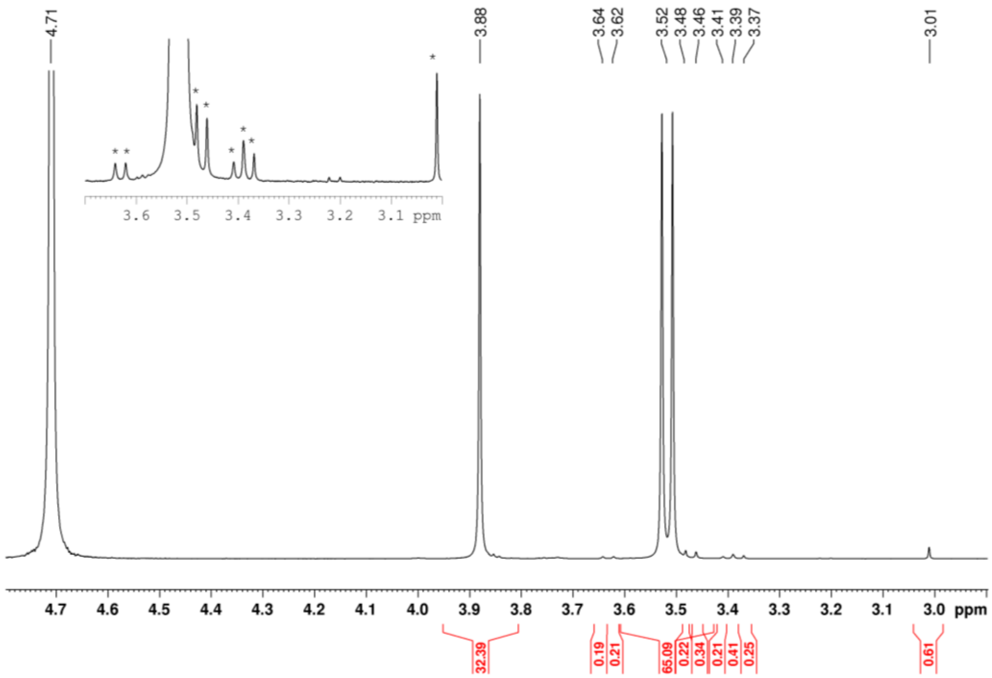

Figure S 3: ^{1}H-NMR-spectrum of EDTMP in D_2O. δ (ppm): 9.23, 12.94. Impurities are marked with "". The sum of integrals is normalized to 100.*

DTPMP

In the ^{31}P-{^{1}H}-NMR-spectrum (Fig. S 4), two main signals in a ratio of 1:4 can be seen, which represent the phosphonate-group in the middle of DTPMP (δ (ppm): 12.94) and the four phosphonate groups of DTPMP attached to the outer amine moieties (δ (ppm): 9.23). Impurities are marked with an asterisk. The sum of all signal-integrals is normalized to 100. Impurities of the analysed DTPMP contribute 1.37 %. The purity of the DTPMP used in the experiments is therefore >98.6 %.

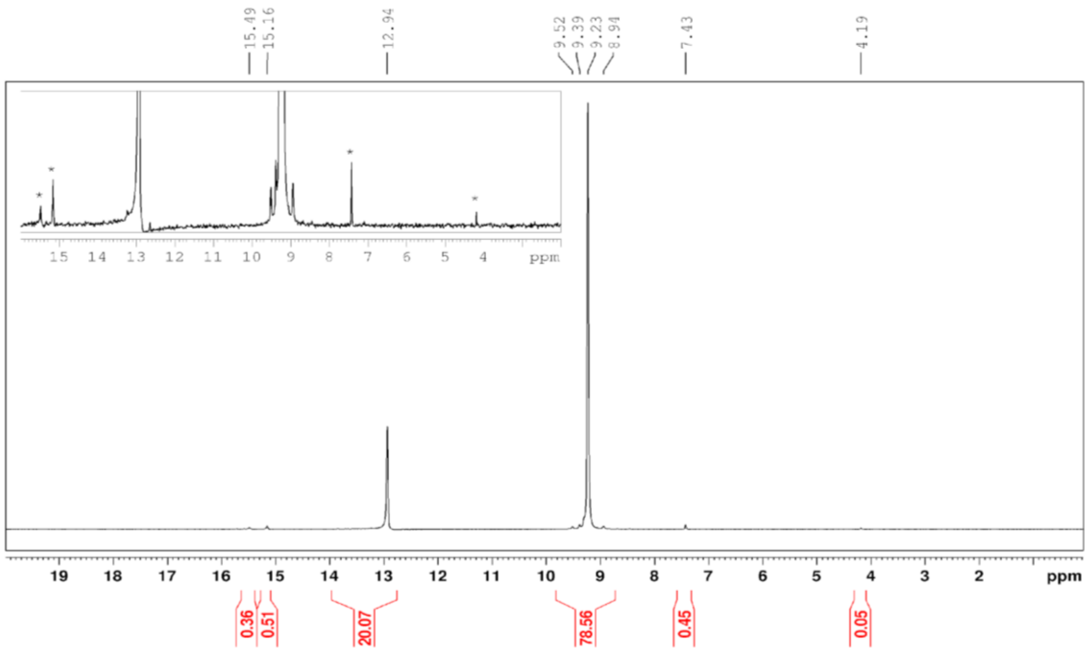

Figure S 4: ^{31}P-{^{1}H}-NMR-spectrum of DTPMP in D_2O. δ (ppm): 9.23, 12.94. Impurities are marked with an asterisk. The sum of integrals is normalized to 100.

Instrumentation

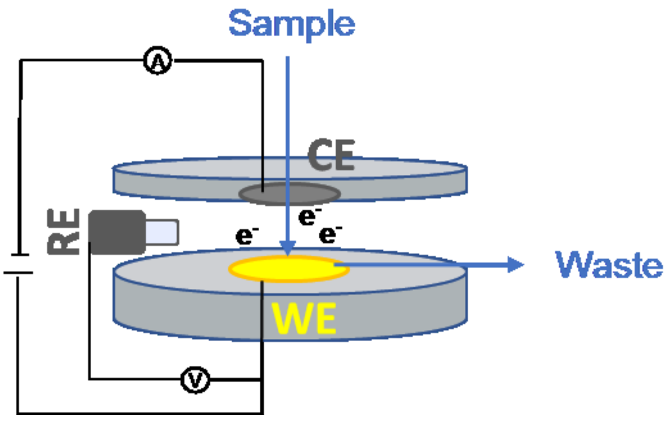

Figure S 5: Schematic depiction of the Wall-Jet cell geometry used for amperometric detection throughout this work. RE = reference electrode, WE = working electrode, CE = counter electrode.

Chromatographic separation

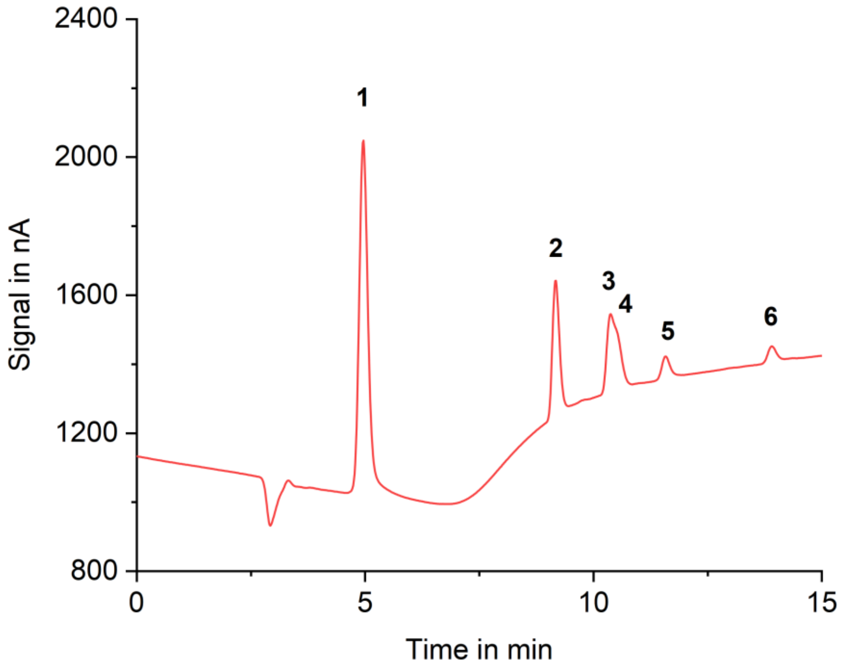

Figure S 6: Chromatogram of a 10 µM multi-phosphonate standard using pure NaOH eluents. Chromatographic conditions: column: Thermo Scientific Dionex AS16 (2x5 + 2x250mm), 30 °C; eluents: A 10 mM NaOH, B 150 mM NaOH, 30 °C, (no column regeneration with 300 mM NaOH for months); gradient profile: B 0-17 min 20-80 % B, 17-20 min 80 % B; 20.1-40 min 0 % B; detection: amperometric detection with gold WE, Pt CE and AG/AgCl RE, waveform as depicted in Figure 5 ***3****) with* E_2 *= 0.27 V; injection volume: 50 µL; 10 µM of 1 = AMPA, 2 = glyphosate, 3 = IDMP, 4 = ATMP, 5 = EDTMP, 6 = DTPMP.*

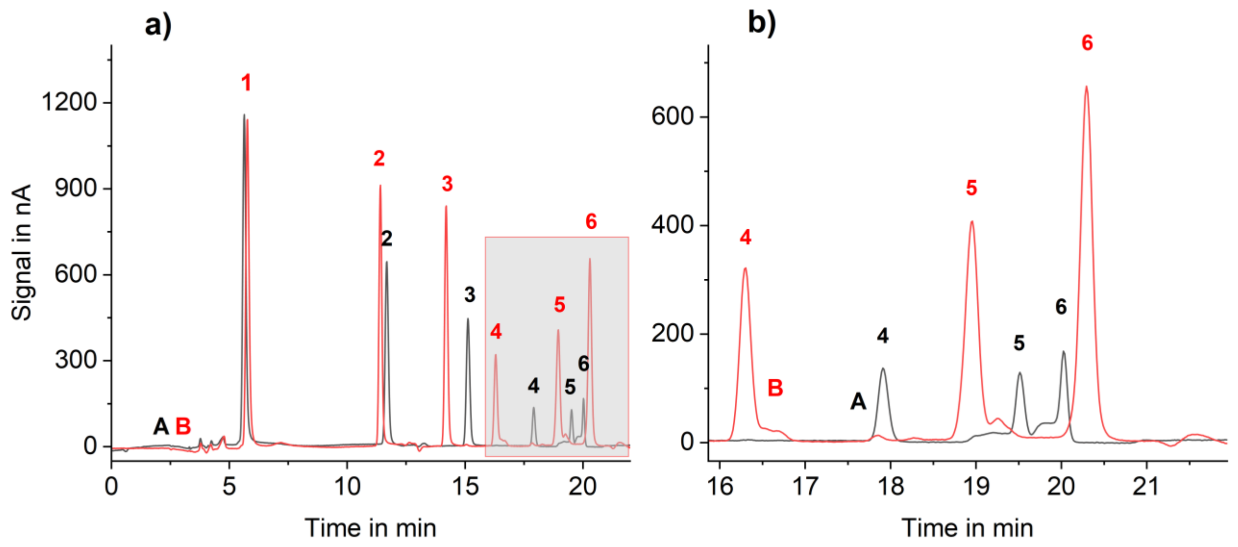

Figure S 7: Two chromatograms of the same 10 µM multi-standard using different gradient profiles and eluents B. Eluent A consists of 15 mM NaOH in both cases, while eluent B consists of 50 mM NaOH and 400 mM NaOAc for ***A*** *(black) and 15 mM NaOH and 400 mM NaOAc for* ***B*** *(red). The chromatograms are shown after blank subtraction.* ***a****) shows the full chromatogram,* ***b****) displays a magnification of the grey rectangle in* ***a****). The chromatographic conditions for* ***B*** *correspond to those in the caption of Figure 2. Chromatographic conditions for* ***A****: column: Thermo Scientific Dionex AS16 (2x5 + 2x250 mm) at 30 °C; eluents* ***A****: 15 mM NaOH,* ***B****: 50 mM NaOH + 400 mM NaOAc; flow rate: 0.3 mL/min; gradient profile: 0-6 min 0 % B, 6-14 min 10-30 % B, 14-18 min 30-100 % B, 18-19 min 100 % B, 20.1-22 min 0 % B, post run: 8 min with 100 % eluent A at 0.6 mL/min; detection: amperometric detector with gold WE, Pt CE and Ag/AgCl RE, 35 °C; waveform: see Figure 5* ***3****); injection volume: 50 µL; 10 µM of: 1 = AMPA, 2 = glyphosate, 3 = IDMP, 4 = ATMP, 5 = EDTMP, 6 = DTPMP.*

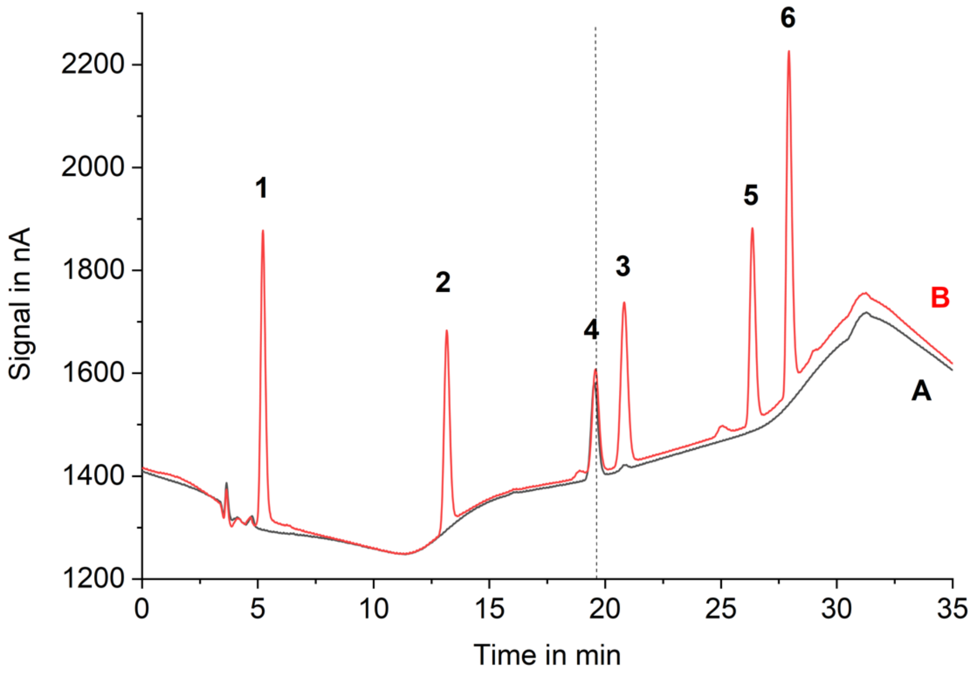

*Figure S 8: Chromatograms of **A** a single ATMP standard and **B** a multi-phosphonate standard. Chromatographic conditions: column: Thermo Scientific Dionex AS16 (2x5 + 2x250 mm) at 30 °C, eluents: A 20 mM NaOH, B 50 mM NaOH + 400 mM NaOAc, 30 °C, (no column regeneration with 300 mM NaOH for months); gradient profile: 0-5 min 10 % B, 5-13 min 10-30 % B, 17-30 min 30-100 % B, 17-20 min 100 % B, 20.1-40 min 0 % B; detection: amperometric detection with gold WE, Pt CE and AG/AgCl RE, waveform as depicted in Figure 5 **3**) with E_2 = 0.25 V; injection volume 50 µL. 10 µM of 1 = AMPA, 2 = glyphosate, 3 = IDMP, 4 = ATMP, 5 = EDTMP, 6 = DTPMP.*

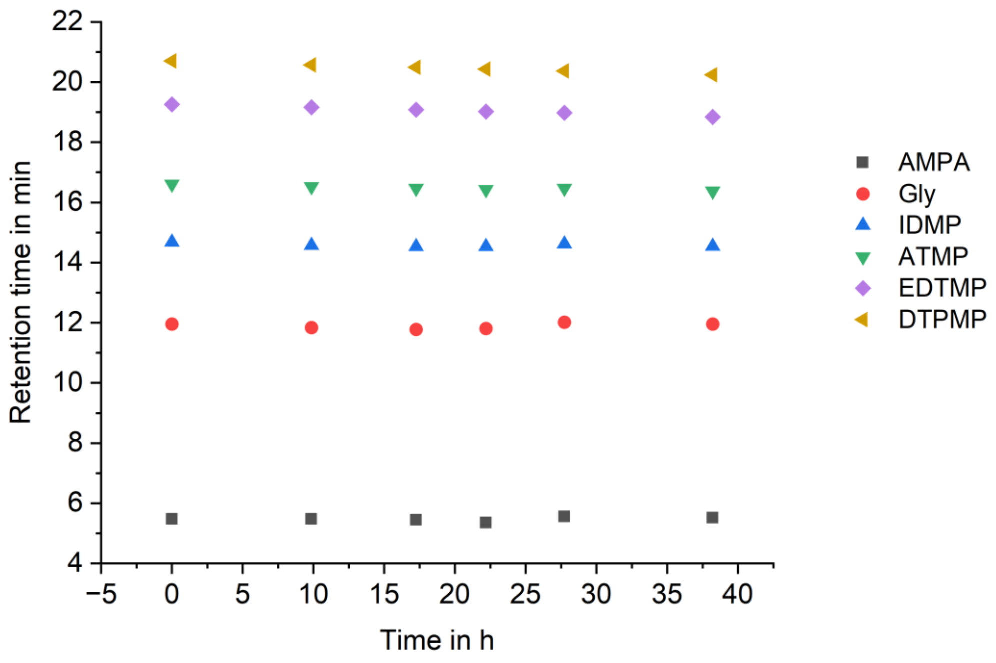

Figure S 9: Retention time of the six investigated analytes in repetitive measurements of a 1 µM multi-standard over 38 hours using the final optimized method presented in the text. Chromatographic conditions: see caption of Figure 2.

(Integrated) pulsed amperometric detection

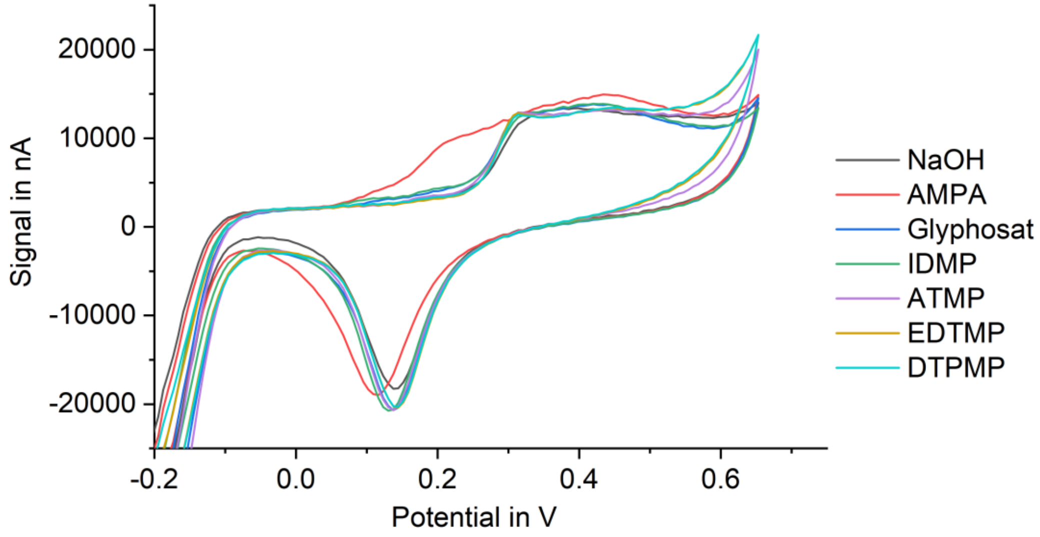

Figure S 10: Cyclovoltammograms of 0.5 mM of the respective aminophosphonate in 0.1 M NaOH background solution, next to the pure background solution.

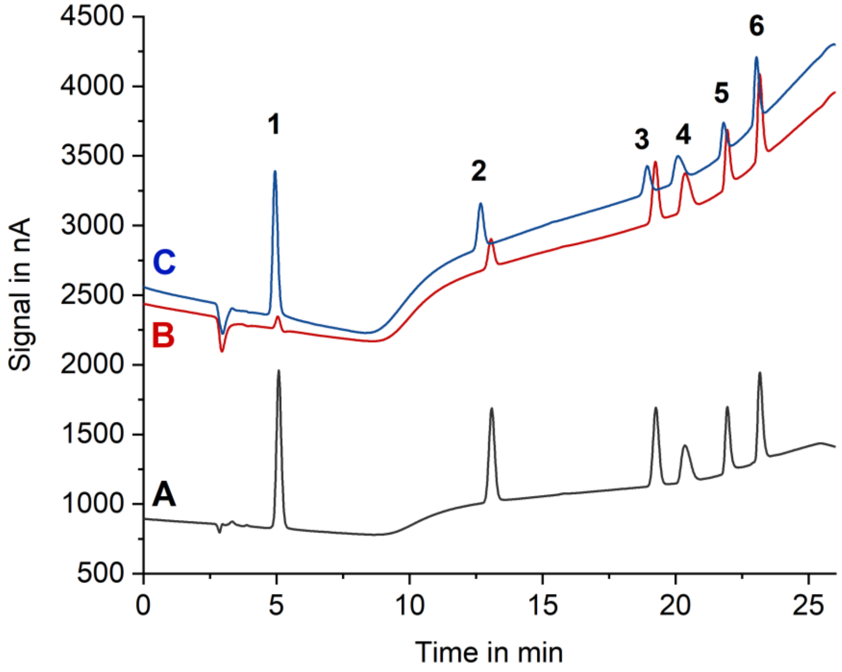

*Figure S 11: Chromatogram recorded using IPAD (**A**) compared to two chromatograms recorded using PAD (**B** & **C**). Chromatographic conditions: **A**, **B** & **C**: Column: Thermo Scientific Dionex AS16 (2x5 + 2x250mm), 30 °C; eluents: A: 15 mM NaOH, B: 50 mM NaOH + 400 mM NaOAc. Gradient profile:0-5 min 10 % B, 5-13 min 10-30 % B, 13-17 min 30-100 % B, 17-20 min 100 % B, 20.1-40 min 0 % B; detection: amperometric detector with gold WE, Pt CE and Ag/AgCl RE. Waveforms: **A**: IPAD waveform similar to Figure 5 **3**) with $E_2 = 0.27$ V, **B**: PAD waveform similar to Figure 5 **2**) with $E_1 = 0.15$ V, **C**: PAD waveform similar to Figure 5 **2**) with $E_1 = 0.27$ V; compounds: 10 µM of 1 = AMPA, 2 = glyphosate, 3 = IDMP, 4 = ATMP, 5 = EDTMP, 6 = DTPMP; injection volume: 50 µL.*

Screening of detector parameters:

*Table S 3: Detector method changes and resulting peak areas of all six analytes in nA*min. Benchmark method applies an IPAD waveform with E_2 = 0.25 V, as depicted in Figure 5 **3**). PAD indicates, that PAD has been applied as depicted in Figure 5 **2**). If not indicated otherwise, IPAD has been applied. The potential numbers (E_1, E_2, …) for PAD and IPAD are explained in Figure S1 and Table S2.*

**Method change compared to benchmark**	**Area in nA*min**					
	**AMPA**	**glyph**	**IDMP**	**ATMP**	**EDTMP**	**DTPMP**
-	277.12	109.65	106.60	123.75	50.31	102.43
-	256.66	119.15	117.84	149.30	68.03	120.89
E_2 = 0.23 V	212.90	114.68	120.08	157.18	70.13	121.13
E_2 = 0.21 V	133.63	100.78	124.22	167.99	74.50	120.53
E_2 = 0.27 V	270.50	145.32	127.28	155.31	74.12	127.54
t_{int} = 750 ms (only trapez)	292.26	145.53	139.87	147.63	70.93	122.65
Change order of E_3 and E_4	262.78	118.27	120.98	172.09	67.46	114.56
Change order of E_3 and E_4 and change t_{ox} & t_{red} (40 vs 60 ms)	247.22	115.74	110.14	149.08	56.56	98.09
Change order of E_3 and E_4 and t_{ox} & t_{red} both 50 ms	261.51	109.14	110.67	158.52	59.02	104.66
PAD, E_1=0.15	22.02	56.60	111.60	103.04	89.89	135.96
PAD, E_1=0.27	237.69	90.24	52.81	70.98	50.49	103.01

Table S 4: Exact potentials and times of the final optimized IPAD waveform.

**Time in ms**	**Start potential in V**	**End potential in V**	**Integration**
0 - 370	0.0	0.0	
370 - 520	0.0	0.0 – 0.27	Start
520 - 630	0.27	0.27	
630 - 750	0.27	0.0	End
750 - 800	0.0	0.0	
800 - 840	-1.0	-1.0	
840 - 900	0.6	0.6	

Greenness

To ensure the highest possible comparability between the three methods and transparency, all criteria have been assigned with the default weight of 2, except for the **principle 1** “Direct analytical techniques” and **3** “In-situ measurements”, which were assigned the weight 1. As all compared methods are off-line methods conducting batch analysis, **principles 1** and **3** are irrelevant for this comparison. The second point that needs clarification concerns **principle 8** “Number of detectable analytes in a single run”: One major advantage of the presented IC-IPAD method is its ability to measure all six analytes presented above – AMPA, glyphosate, IDMP, ATMP, EDTMP, and DTPMP – in the same chromatographic run. While existing polyphosphonate methods sometimes included other substances, e.g. HEDP, PBTC or sulfate, in this work we solely focus on the six substances mentioned above. Thus, if a method is able to quantify sulfate, nitrate, PBTC and EDTMP, the “number of detectable analytes” will be counted one (EDTMP) in this comparison. Last but not least the “amount of sample needed” (**principle 2**) needs clarification. Due to the different concentration ranges of the samples measured in the three methods (natural samples vs. laboratory batch experiments) and the required preconcentration steps for natural samples, the “amount of sample needed” was not comparable. Therefore, the injection volumes were drawn for comparison instead.

Application example: DTPMP oxidation by MnO_2

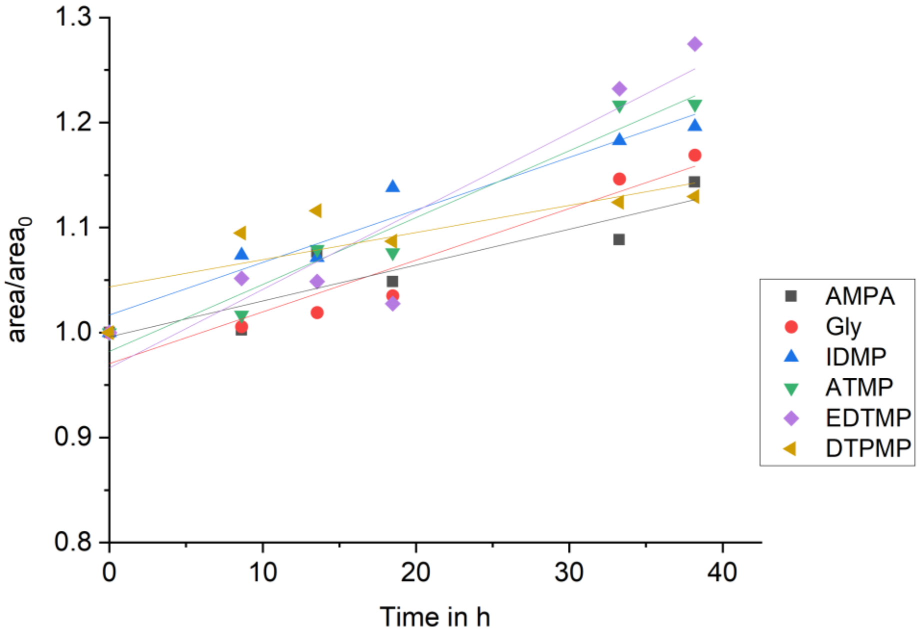

Figure S 12: Normalized peak areas of the six analytes in 1 µM multi-phosphonate check standards over time. The peak areas are all normalized to the peak area of the respective analyte measured at timepoint zero. From the linear regression (see below) a correction factor – relative increase per hour – has been calculated for each analyte, which was then applied to the sample measurements, see equation below.

Correction of sample concentrations:

The corrected concentration is derived by subtracting the product of correction factor (*f*), time (*t*) in hours, and initially calculated concentration (c_{int}) from the initially calculated concentration.

$$c_{corr} = c_{init}(1 - f \cdot t)$$

Table S 5: Calculated correction parameters from the 1 µM check standards shown in Figure S 9.

Analyte	Relative increase after 38 hours	r^2 of linear regression	Correction factor f (rel. increase/h)
AMPA	1.143404	0.8274	0.003755
Glyphosate	1.168947	0.9218	0.004424
IDMP	1.196055	0.9434	0.005134
ATMP	1.217679	0.9563	0.005701
EDTMP	1.274751	0.8727	0.007195
DTPMP	1.129571	0.6225	0.003393

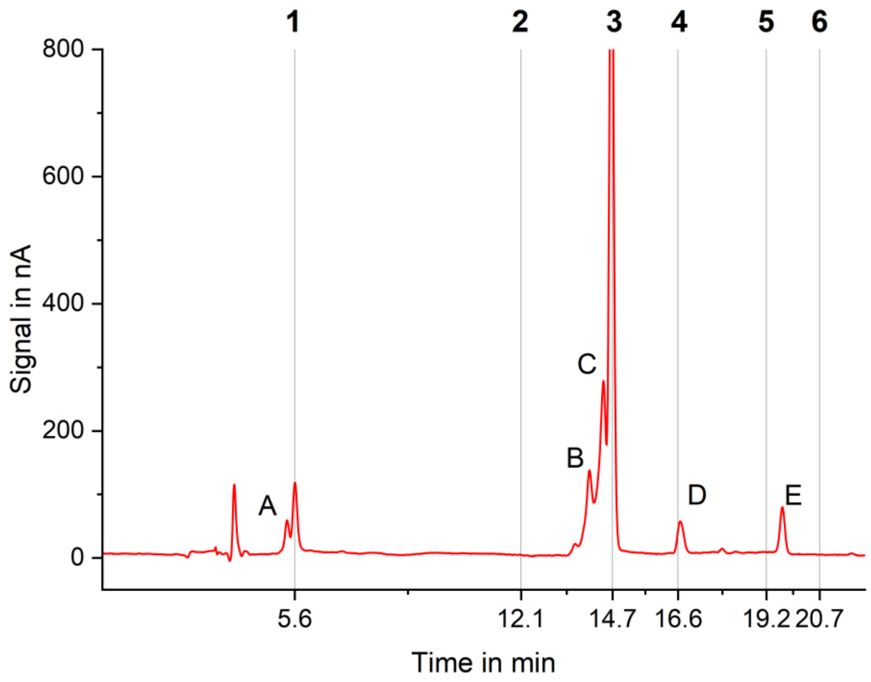

Figure S 13: Chromatogram of the aqueous phase of the DTPMP/MnO_2 transformation experiment after 3.0 hours applying blank subtraction. For better depiction of the low concentrated TPs, the y-axis maximum was decreased below the IDMP peak height. Unknown TPs were assigned the letters A-E. Chromatographic conditions as described in the caption of Figure 2. Compounds: 1 = AMPA, 2 = glyphosate, 3 = IDMP, 4 = ATMP, 5 = EDTMP, 6 = DTPMP; injection volume: 50 µL.

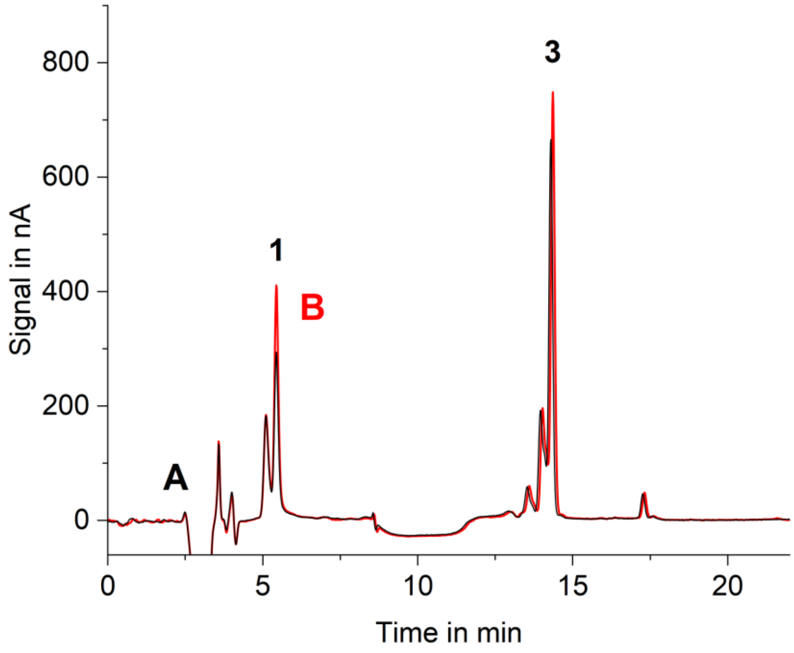

*Figure S 14: Chromatograms of the sorbed fraction of the sampling point after 0.67 h, with (**A**) and without (**B**) standard addition of 1 µM AMPA (**1**) and IDMP (**2**). Chromatographic conditions as described in the caption of Figure 2.*

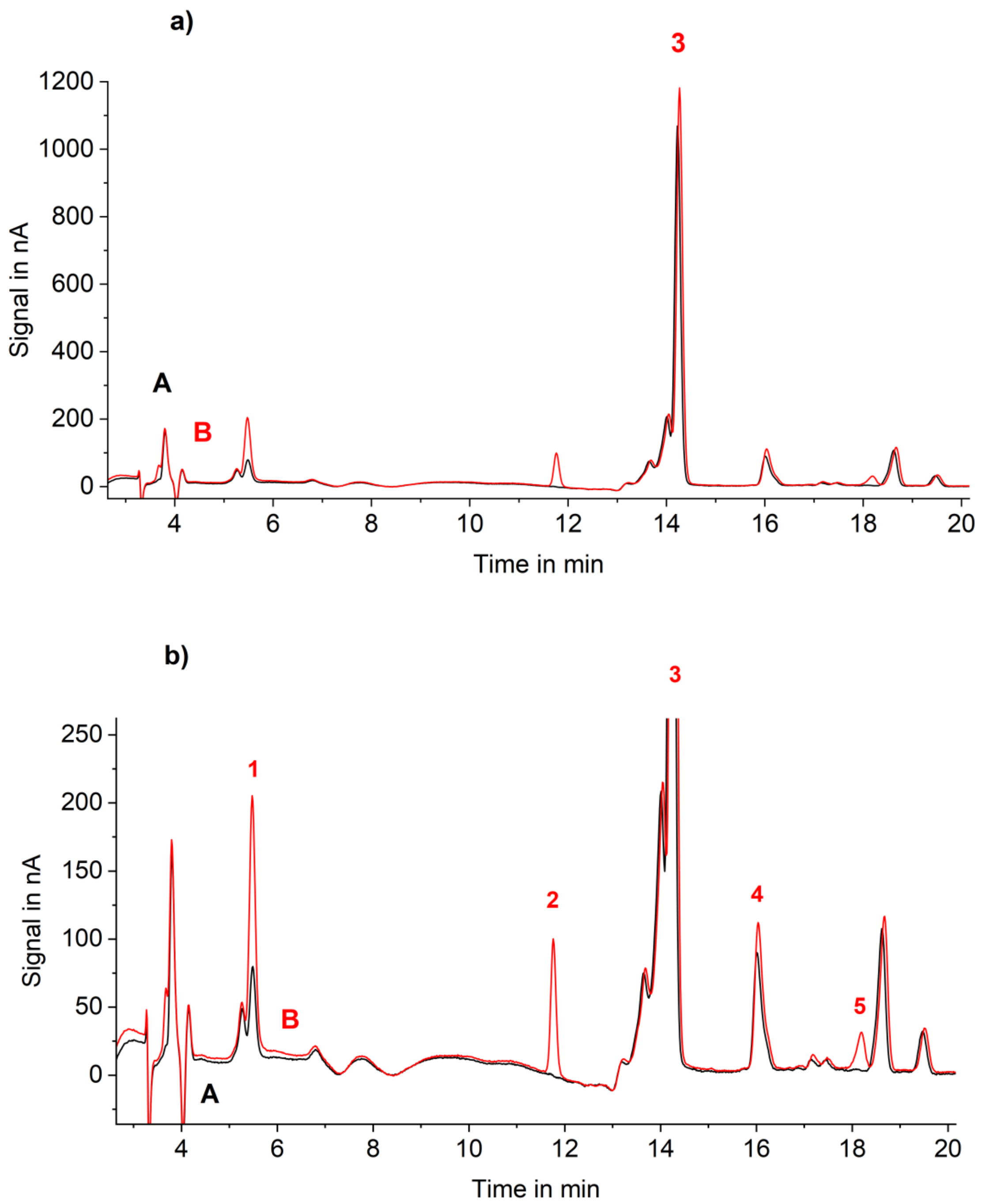

Figure S 15: Chromatograms of the aqueous phase of the DTPMP/MnO_2 transformation experiment after 3.0 hours applying blank subtraction. ***A*** *shows the pure sample, while* ***B*** *shows a sample with standard addition of 1 µM AMPA (**1**), glyphosate (**2**), IDMP (**3**), ATMP (**4**), and EDTMP (**5**).* ***a)*** *shows the full chromatogram, while* ***b)*** *shows y-axis magnification. Chromatographic conditions as described in the caption of Figure 2.*

References

1. Wang S, Sun S, Shan C, Pan B. Analysis of trace phosphonates in authentic water samples by pre-methylation and LC-Orbitrap MS/MS. Water Res. 2019 Sep 15;161:78–88.

2. Fürhacker M, Lesueur C, Pfeffer M, Köllensperger G, Popp M, Mentler A. Phosphonate - AMPA (Aminomethylphosphonsäure). Herkunftsabschätzung, Umweltkonzentrationen und Photolyseabbau. Vienna; 2005 Dec.

3. Schmidt CK, Raue B, Brauch HJ, Sacher F. Trace-level analysis of phosphonates in environmental waters by ion chromatography and inductively coupled plasma mass spectrometry. Int J Environ Anal Chem. 2014 Mar;94(4):385–98.

4. Armbruster D, Müller U, Happel O. Characterization of phosphonate-based antiscalants used in drinking water treatment plants by anion-exchange chromatography coupled to electrospray ionization time-of-flight mass spectrometry and inductively coupled plasma mass spectrometry. J Chromatogr A. 2019 Sep 13;1601:189–204.

5. Klinger J, Sacher F, Brauch HJ, Maier D. Determination of Organic Phosphonates in Aqueous Samples Using Liquid Chromatography/ Particle-beam Mass Spectrometry. Acta hydrochim hydrobiol. 1997;25(2):79–86.

6. Tewari MJK, van Stroe-Bieze S. Analysis of amine-containing phosphonates in detergent powders by anion-exchange chromatography with pulsed amperometric detection. J Chromatogr A. 1997;771:155–61.

7. Wong D, Jandik P, Jones WR, Hagenaars A. Ion chromatography of polyphosphonates with direct refractive index detection. J Chromatogr. 1987;389:279–85.

8. Weiss J, Hägele G. Ionen-chromatographische Analyse anorganischer und organischer Komplexbildner. Fresenius Z Anal Chem. 1987;348:46–50.

9. Nowack B. Determination of phosphonates in Natural Waters by ion-pair high-performance Liquid Chromatography. J Chromatogr A. 1997;773:139–46.

10. Vaeth E, Sladek P, Kenar K. Ionen-Chromatographie von Polyphosphaten und Phosphonaten. Fresenius Z Anal Chem. 1987;329.

11. Weiss J. Handbook of Ion Chromatography. 4th ed. Weinheim: Wiley-VCH; 2016.

12. Fedorowski J, LaCourse WR. A review of pulsed electrochemical detection following liquid chromatography and capillary electrophoresis. Anal Chim Acta. 2015 Feb 25;861:1–11.

13. LaCourse WR. Pulsed Electrochemical Detection in High Performance Liquid Chromatography. 1st ed. New Jersey: Wiley & Sons Ltd; 1997.

14. Oromí-Farrús M, Minguell JM, Oromi N, Canela-Garayoa R. A Reliable Method for Quantification of Phosphonates and Their Impurities by ^{31} P NMR. Anal Lett. 2013 Aug 13;46(12):1910–21.
